# Quadratic Sparse Domination and Weighted Estimates for Non-integral Square Functions

**DOI:** 10.1007/s12220-022-01031-w

**Published:** 2022-11-14

**Authors:** Julian Bailey, Gianmarco Brocchi, Maria Carmen Reguera

**Affiliations:** School of Mathematics, University of Birmingham, Edgbaston, Birmingham, B15 2TT UK

**Keywords:** Elliptic operator, Sparse bounds, Sharp weighted estimates, Square functions, 42B20, 42B37

## Abstract

We prove a quadratic sparse domination result for general non-integral square functions *S*. That is, for $p_0 \in [1,2)$ and $q_0 \in (2,\infty ]$, we prove an estimate of the form where $q_{0}^{*}$ is the Hölder conjugate of $q_{0}/2$, *M* is the underlying doubling space and ${\mathcal {S}}$ is a sparse collection of cubes on *M*. Our result will cover both square functions associated with divergence form elliptic operators and those associated with the Laplace–Beltrami operator. This sparse domination allows us to derive optimal norm estimates in the weighted space $L^{p}(w)$.

## Introduction

Recent years have seen a surge of activity in the weighted theory of singular integrals that has resulted in the resolution of some major conjectures such as the $A_2$ conjecture [30], the Muckenhoupt–Wheeden conjecture [48] and the resolution of the two weight problem for the Hilbert transform [34, 36]. Accompanying these achievements is the development of new core techniques such as the representation of singular integrals by dyadic operators [30] or the sparse domination of singular integrals [17, 43].

The sparse domination of an operator provides, at a glance, a rich picture of the unweighted and weighted estimates with precise tracking of the dependence on the weight characteristic. Its use in harmonic analysis was introduced by Lerner in [39], where a decomposition of an arbitrary measurable function was obtained in terms of its local mean oscillations. It has since been extended to a great number of different contexts spanning and reproducing a large portion of harmonic analysis. To call attention to some of the most celebrated results, there have been articles published covering the domination of Calderón–Zygmund singular integral operators [17, 35, 41–43], multilinear singular integrals [21], rough singular integrals, variational Carleson, Bochner–Riesz multipliers, Walsh–Fourier multipliers, spherical maximal function and also the *T*1 sparse domination of singular integrals. For more details on these and other applications we refer the reader to Sect. 8 of the survey paper [47] and the references therein. In this article we are interested in the sparse domination of square function operators.

The sparse domination of classical square function operators was first considered in [40]. In this article it was discovered that in order to obtain sharp weighted estimates for square functions from a sparse domination result, the sparse techniques applied to singular integral operators had to be adjusted to account for the quadratic nature of the square function. Thus, instead of a “linear” sparse domination result, one must aim for a stronger “quadratic” sparse domination theorem. This idea was also explored in [11] where a quadratic result with minimal *T*1-type assumptions is proved. Similar ideas are also investigated in the work of Lorist [44], where sparse domination is obtained for general vector-valued operators.

Since the turn of the century, fuelled by applications to boundary value problems and the epic contest of ideas surrounding the Kato conjecture, there has been a sustained and pronounced interest in weighted estimates for non-integral singular operators that are beyond the realm of Calderón–Zygmund theory. Some of the most prominent examples are operators attached to the divergence form elliptic operator $L = - \mathrm {div} (A \nabla )$, where *A* is bounded and elliptic with complex coefficients. For instance, neither the Riesz transforms $\nabla L^{-\frac{1}{2}}$ nor the constituent operators $\{\sqrt{t} \nabla e^{-t L}\}_{t>0}$ of the square function

1.0.1 $$\begin{aligned} G_{L} f = \left( \int ^{\infty }_{0} \left|\sqrt{t} \nabla e^{-t L} f \right|^{2} \frac{ \, {\text {d}}{t}}{t} \right) ^{1/2} \end{aligned}$$

possess integral kernels in general that satisfy any meaningful estimates and, as such, are deserving of the title “non-integral”. As a result of this characteristic, and in contrast to the classical setting of the Laplacian operator $\Delta$, these operators will fail to be bounded on $L^{p}({\mathbb {R}}^{n})$ for *p* in the entire interval range $(1,\infty )$. Instead, as proved in [1], boundedness will occur if and only if *p* is contained within a restricted subinterval of $(1,\infty )$ that will depend on the perturbation *A*, see also [9] and [28]. Similarly, for boundedness on the weighted space $L^{p}(w)$, one must also consider a restricted range of $p \in (1,\infty )$. For a detailed investigation into such results the reader is referred to the seminal series of papers by P. Auscher and J. M. Martell, [2–5].

The sparse domination methods developed for Calderón–Zygmund operators in [35, 41, 42] automatically imply boundedness on $L^{p}({\mathbb {R}}^{n})$ for *p* in the full range $(1,\infty )$. It then follows that the classical sparse domination is particularly ill-suited to non-integral singular operators. In the article [8], the authors F. Bernicot, D. Frey and S. Petermichl introduced a linear sparse domination framework that was adapted to non-integral singular operators in the sense that the sparse object dominating the operator would only be bounded on a restricted range. This linear sparse domination allowed for sharp weighted estimates to be produced for a wide range of operators associated with *L* that included the Riesz transforms $\nabla L^{-\frac{1}{2}}$.

As stated earlier for the classical setting of the Laplacian, the linear sparse domination in [8] does not imply the best weighted bounds for square functions for $p>2$. The ultimate objective of this article is, thus, to prove a quadratic sparse domination theorem for non-integral square functions. This, in turn, will yield weighted estimates for $G_{L}$ and other similar square functions. They will also reproduce optimal weighted estimates for $G_{L}$ when $L = - \mathrm {div}(A \nabla )$ and *A* is real valued with smooth coefficients, a result that was first proved by T. A. Bui and X. Duong in [13]. When the square function is bounded in the full range $(1,\infty )$, we recover the sparse form in [11] which implies weighted estimates that are known to be optimal for several classical square functions [40].

Motivated by finding a uniform setting that will include several examples of square functions, we consider the following general framework. The underlying space $(M,d,\mu )$ is a locally compact separable metric space (*M*, *d*) equipped with a Borel measure $\mu$ that is finite on compact sets and strictly positive on any non-empty open set. For a measurable subset $B \subset M$, we denote $|B |:=\mu (B)$.

The measure $\mu$ will be assumed to satisfy the doubling property,

1.0.2 $$\begin{aligned} \left|B(x,2 r) \right| \lesssim \left|B(x,r) \right| \end{aligned}$$

for all $x \in M$ and $r > 0$, where *B*(*x*, *s*) denotes the ball of radius $s > 0$ centred at a point $x \in M$ and $X \lesssim Y$ will be used throughout the paper to signify that there exists a constant $C>0$ such that $X \le C Y$.

There will then exist some $\nu > 0$ for which

1.0.3 $$\begin{aligned} \left|B(x,r) \right| \lesssim \left( \frac{r}{s} \right) ^{\nu } \left|B(x,s) \right| \qquad \forall \, x \in M, \, r \ge s > 0. \end{aligned}$$

It will be assumed that there exists some non-decreasing function $\varphi : (0,\infty ) \rightarrow (0,\infty )$ with $\varphi (1) = 1$ for which

1.0.4 $$\begin{aligned} \left|B(x,r) \right| \simeq \varphi \left( \frac{r}{s} \right) \left|B(x,s) \right| \end{aligned}$$

for all $x \in M$ and $r, \, s > 0$, where $X \simeq Y$ means that both $X \lesssim Y$ and $Y \lesssim X$ hold. This technical condition has been imposed in order to prove boundedness of a certain maximal operator that is essential to our proof. This point will be elaborated upon further in Remark 1.4 and Sect. 4.

Let $\omega \in [0,\pi /2)$. We say that a linear operator *L* with dense domain ${\mathcal {D}}_2(L)$ in $L^2(M,\mu )$ is $\omega$-accretive if its spectrum is contained in the closed sector $\Sigma _{\omega ^+} :=\{ z \in {\mathbb {C}}\,:\, |\arg z |\le \omega \} \cup \{ 0 \}$ and $\langle L f,f \rangle \in \Sigma _{\omega ^+}$ for all *f* in ${\mathcal {D}}_2(L)$.

We will consider an unbounded operator *L* on $L^{2}(M,\mu )$ satisfying the below assumption.

### Assumption 1.1

*L* is an injective linear operator on $L^{2}(M,\mu )$ with dense domain ${\mathcal {D}}_{2}(L) \subset L^{2}(M,\mu )$. *L* is $\omega$-accretive for some $0 \le \omega < \pi / 2$ and there exists some $1 \le p_{0}< 2 < q_{0} \le \infty$ and $c > 0$ such that for all balls $B_{1}, \, B_{2}$ of radius $\sqrt{t}$,

$$\begin{aligned} \left\Vert e^{-t L} \right\Vert _{L^{p_{0}}(B_{1}) \rightarrow L^{q_{0}}(B_{2})} \lesssim \left|B_{1} \right|^{-\frac{1}{p_{0}}} \left|B_{2} \right|^{\frac{1}{q_{0}}} e^{-c \frac{d(B_{1},B_{2})^{2}}{t}}. \end{aligned}$$

From Assumption 1.1, it follows that *L* is a maximal accretive operator on $L^{2}(M,\mu )$, *L* possesses a bounded holomorphic functional calculus on $L^{2}(M,\mu )$ and $-L$ is the generator of an analytic semigroup $(e^{-t L})_{t > 0}$ on $L^{2}(M,\mu )$.

Throughout this article, we consider square function operators associated with *L*. These will be defined to be operators *S* that satisfy the following set of assumptions.

### Assumption 1.2

- The operator *S* is sublinear and bounded on $L^{2}(M,\mu )$.
- *(Off-diagonal estimates for the constituent operators)* The operator *S* is of the form
  $$\begin{aligned} Sf(x) := \left( \int ^{\infty }_{0} \left|{\mathcal {Q}}_{t}f(x) \right|^{2} \, \frac{ \, {\text {d}}{t}}{t} \right) ^{\frac{1}{2}}, \end{aligned}$$
  where $\left\{ {\mathcal {Q}}_{t} \right\} _{t > 0}$ is a collection of bounded operators on $L^{2}(M,\mu )$ which satisfy the property that there exists some $1 \le p_{0}< 2 < q_{0} \le \infty$ such that for all balls $B_{1}, \, B_{2}$ of radius $\sqrt{t}$,
  $$\begin{aligned} \left\Vert {\mathcal {Q}}_{t} \right\Vert _{L^{p_{0}}(B_{1}) \rightarrow L^{q_{0}}(B_{2})} \lesssim \left|B_{1} \right|^{-\frac{1}{p_{0}}} \left|B_{2} \right|^{\frac{1}{q_{0}}} \left( 1 + \frac{d(B_{1},B_{2})^{2}}{t} \right) ^{-(\nu + 1)}. \end{aligned}$$
- *(Cancellation with respect to L)* There exists $A_{0} > 0$ and $N_{0} \in {\mathbb {N}}$ such that for all integers $N \ge N_{0}$,
  $$\begin{aligned} {\mathcal {Q}}_{t} (s L)^{N} e^{-s L} = \frac{t^{A_{0}} s^{N}}{(t + s)^{A_{0} + N}} \Theta _{t + s}^{(N)}, \end{aligned}$$
  where $\lbrace \Theta _{r}^{(N)} \rbrace _{r > 0}$ is a collection of bounded operators on $L^{2}(M,\mu )$ that satisfies off-diagonal estimates at all scales in the sense that
  $$\begin{aligned} \big \Vert \Theta _{r}^{(N)} \big \Vert _{L^{p_{0}}(B_{1}) \rightarrow L^{q_{0}}(B_{2})} \lesssim \left|B_{1,\sqrt{r}} \right|^{-\frac{1}{p_{0}}} \left|B_{2,\sqrt{r}} \right|^{\frac{1}{q_{0}}} \left( 1 + \frac{d(B_{1},B_{2})^{2}}{r} \right) ^{- \frac{\nu + 1}{2}} \end{aligned}$$
  for all balls $B_{1}, \, B_{2} \subset M$ and $r > 0$, where $B_{i,\sqrt{r}} := (\sqrt{r}/r(B_{i})) B_{i}$ for $i = 1, \, 2$ and for a ball $B = B(x,r)$ and $t > 0$ we will use the notation *tB* to represent the *t*-dilate of *B*, $t B :=B(x,t r)$.
- *(Cotlar type inequality)* There exists an exponent $p_{1} \in [p_{0},2)$ such that for all $x \in M$ and $r > 0$ where we define  for $f\in L^1_{\mathrm {loc}}(M,\mu )$ and we denote by ${\mathcal {M}}$ the uncentered Hardy–Littlewood maximal function and ${\mathcal {M}}_pf :=({\mathcal {M}} |f|^p )^{1/p}$ for any $p\ge 1$.

### Remark 1.3

In general, the exponents $p_0$ and $q_0$ are determined by the off-diagonal estimates for the constituent operator ${\mathcal {Q}}_t$, rather than by the off-diagonal estimates for $\{e^{-tL}\}_{t>0}$.

For our aim, it is enough to assume that the range in which one has off-diagonal estimates for $\{e^{-tL}\}_{t>0}$ contains the range $(p_0,q_0)$ in the Assumption 1.2.

### Remark 1.4

As our work is intended to build upon the article [8], it will be instructive to compare our assumptions with the hypotheses of [8]. In both our article and [8], the assumptions imposed upon the underlying operator *L* are identical. For the operator *S*, we have also assumed $L^{2}$-boundedness and a Cotlar type inequality. However, we have included the additional assumption that *S* is of the form of a square function composed of operators ${\mathcal {Q}}_{t}$ that satisfy off-diagonal bounds. Also, the cancellative condition of *S* with respect to *L*, Assumption (b) of [8], has instead been replaced by a cancellative condition of the constituent operators ${\mathcal {Q}}_{t}$.

In Sect. 4, using the growth condition imposed upon our metric space (1.0.4), it will be proved that the assumed cancellative condition for the ${\mathcal {Q}}_{t}$ operators does in fact imply the cancellative condition of *S* with respect to *L*. This allows us to deduce that the operators under consideration in our article are a restricted subclass of the operators considered by [8]. Indeed, the additional growth condition of our metric space (1.0.4) has been assumed with the sole purpose of ensuring that we are working strictly within the setting of [8]. This will allow us to utilise some of the intermediary results from [8] without having to reprove them under a different cancellation condition. This will be of particular use to us in Sect. 4 when we come to prove the boundedness of a certain maximal function operator that is essential to our proof.

### Remark 1.5

One does not have to search for long before encountering examples of square function operators that satisfy the previous set of assumptions. For instance, the square functions associated with an elliptic operator $L = - \mathrm {div} A \nabla$, such as $G_{L}$ from (1.0.1) and

$$\begin{aligned} g_{L}f :=\left( \int ^{\infty }_{0} \left|t L e^{-t L}f \right|^{2} \frac{ \, {\text {d}}{t}}{t} \right) ^{\frac{1}{2}}, \end{aligned}$$

and square functions associated with the Laplace–Beltrami operator satisfy the above conditions. We discuss these examples in detail in Sect. 3.

In order to make sense of the concept of sparse domination and precisely state our main theorem we need to define the notion of a sparse family of cubes. We consider a system of dyadic cubes $\mathscr {D}$ on the metric space (*M*, *d*).

### Definition 1.6

A collection of dyadic cubes ${\mathcal {S}} \subseteq \mathscr {D}$ is $\frac{1}{2}$-sparse if there exists a disjoint collection of sets $\{ F_P \,:\, P \in {\mathcal {S}}\}$ such that for every $P \in {\mathcal {S}}$ we have $F_{P} \subset P$ and $|F_P|> \frac{1}{2} |P|$.

### Theorem 1.7

Let $p_0<2<q_0$ and consider operators *L* and *S* that satisfy Assumptions 1.1 and 1.2 for this choice of exponents. For any *f* and *g* in $C^\infty _c(M)$ there exists a sparse family ${\mathcal {S}} \subseteq \mathscr {D}$ such that1.0.5where $q_{0}^* :=\left( \frac{q_{0}}{2} \right) '$ is the dual exponent of $\frac{q_0}{2}$, and *c* is a positive constant independent of *f* and *g*.

The right hand side of (1.0.5) is the sparse form natural to the square function. We observe that the bilinear sparse form obtained differs from the linear sparse domination results where the $L^{q_{0}'}$ average of *g* is used instead (c.f. [8]). This is due to the non-linear nature of the problem at hand. Analogous sparse forms appear when controlling vector-valued operators, as seen in the work of Lorist [44]. In fact, as the operators we consider satisfy the hypotheses from [8], it follows that [8, Thm. 5.7] will be valid for *S*. This result states that for any *f* and *g* in $C^\infty _c(M)$ there exists a sparse family ${\mathcal {S}} \subseteq \mathscr {D}$ for whichThe essence of our sparse domination result is that, under the additional square function hypotheses assumed above, the previous sparse bound can be improved to a quadratic sparse domination bound that is uniquely suited to square function operators.

Our proof strategy requires the weak boundedness at the endpoint of a“grand maximal function” operator associated with the square function. This strategy is an adaptation of Lerner’s work on singular integrals [42] to our setting, which itself is an elaboration of Lacey’s elementary proof from [35]. The weak-type boundedness of our grand maximal operator will be obtained by demonstrating that our operator is pointwise controlled from above by a related maximal operator that was introduced in [8]. The weak boundedness of this alternative grand maximal operator was proved in [8] under their setting. Since, as will be shown in Sect. 4, we are working strictly within their setting, this will then allow us to conclude that our grand maximal operator is also weakly bounded at the endpoint.

Next we give an account of the weighted estimates that we obtain for our square functions via the sparse domination (Theorem 1.7). It is understood that if the operator at hand maps $L^p$ to $L^p$ for a restricted range of exponents *p*, the relevant classes of weights will involve the intersection of Muckenhoupt and reverse Hölder weights [3]. We define them precisely.

A weight *w* is a positive locally integrable function. We say that a weight *w* is in the Muckenhoupt $A_p$ class for $1<p<\infty$ and we denote it by $w\in A_p$ if and only ifwhere $p'=p/(p-1)$ is the dual exponent of *p*. We say that a weight *w* belongs to the reverse Hölder class $RH_p$ for $p>1$ ifWe can now state our second result.

### Theorem 1.8

Fix $p_0<2<q_0$. For any sparse family ${\mathcal {S}} \subset \mathscr {D}$, functions $f,\,g \in L^{1}_{{\text {loc}}}( \, {\text {d}}{\mu })$, $p \in (2,q_{0})$ and weight $w\in A_{\frac{p}{p_{0}}}\cap RH_{\left( \frac{q_{0}}{p}\right) ^{'}}$ we havewhere

$$\begin{aligned} \gamma (p) :=\max \left( \frac{1}{p - p_{0}}, \left( \frac{q_{0}}{p} \right) ' \frac{1}{2q_{0}^{*}} \right) \,\,\, \text { and } \,\,\, \sigma :=w^{1-p^{*}}. \end{aligned}$$

The constant $C_0$ is independent of both the weight and the sparse collection, and the dependence of this estimate on the weight characteristic is sharp.

Expanding further upon the above theorem, the result is sharp in the sense that the dependence on the weight characteristic $\left[ w \right] _{A_{p/p_{0}}} \left[ w \right] _{RH_{(q_{0}/p)'}}$ can be matched at least asymptotically with the right choice of functions, weights and sparse form. A detailed proof of this sharpness will be presented in Sect. 7. The above theorem, when combined with our other main result, Theorem 1.7, allows us to obtain as a corollary the following sharp weighted result for non-integral square functions. It is important to note that the combination of Theorems 1.7 and 1.8 only produces the below weighted bounds for $p \in (2, q_{0})$. The weighted estimates for the full range $p \in (p_{0},q_{0})$ follows from this on applying a quantitative version of the limited range extrapolation theorem by Auscher and Martell in [3, Thm. 4.9]. See also [46, Thm. 2.2].

### Corollary 1.9

Let $p_0<2<q_0$ and consider operators *L* and *S* that satisfy Assumptions 1.1 and 1.2 for this choice of exponents. For $p \in (p_{0},q_{0})$ and $w \in A_{\frac{p}{p_{0}}} \cap RH_{\left( \frac{q_{0}}{p} \right) '}$ the square function *S* is bounded on $L^{p}(w)$ with

1.0.6 $$\begin{aligned} \left\Vert S \right\Vert _{L^{p}(w)} \lesssim \left( \left[ w \right] _{A_{\frac{p}{p_{0}}}} \cdot \left[ w \right] _{RH_{(\frac{q_{0}}{p})'}} \right) ^{\gamma (p)}, \end{aligned}$$

where $\gamma (p)$ is as defined in Theorem 1.8.

The result is sharp for certain square functions, see [13, 38, 40]. Sharpness can be deduced from the asymptotic behaviour of the unweighted estimates [26]. Unfortunately, these asymptotics are not easy to exactly compute for our non-integral square functions. However, the estimate (1.0.6) implies an upper bound on the asymptotic behaviour of the unweighted norm $\Vert S \Vert _{L^p \rightarrow L^p}$, see Sect. 7.1. In particular, when such asymptotic behaviour is known to match the upper bound, the weighted estimates in Corollary 1.9 are sharp.

### Structure of the Paper

The paper is distributed as follows. Section 2 contains some preliminary results that will be of use later in the paper. Section 3 will discuss the examples that fit the assumptions and that one should keep in mind as references. The proof of Theorem 1.7 requires us to understand the boundedness properties of a grand maximal operator associated with the corresponding square functions. These boundedness properties are included in Sect. 4. Section 5 is dedicated to the proof of our main result, Theorem 1.7. Section 6 considers weighted estimates for the sparse forms found in Sect. 5 and, in particular, proves Theorem 1.8. Finally, Sect. 7 is dedicated to the proof of the sharpness of Theorem 1.8 when $p>2$.

## Preliminaries

In this section we gather a collection of useful results concerning dyadic analysis in metric measure spaces, off-diagonal estimates for a family of operators, and properties of Muckenhoupt and reverse Hölder weight classes.

### Dyadic Analysis on a Doubling Metric Space

We recall some well-known definitions and facts from dyadic harmonic analysis as written in [8]. For detailed information on the construction of dyadic systems of cubes in doubling metric spaces, the interested reader is referred to [31] and references therein.

#### Definition 2.1

A dyadic system of cubes in a metric measure space $(M, \mu )$, with parameters $0< c_{0} \le C_{0} < \infty$ and $\delta \in (0,1)$, is a family of open subsets $\left( Q^{l}_{\alpha } \right) _{\alpha \in {\mathcal {A}}_{l}, l \in {\mathbb {Z}}}$ that satisfies the following properties:

- For each $l \in {\mathbb {Z}}$, there exists a subset $Z_{l}$ with $\mu (Z_{l}) = 0$ such that
  $$\begin{aligned} M = \bigsqcup _{\alpha \in {\mathcal {A}}_{l}} Q^{l}_{\alpha } \bigsqcup Z_{l}; \end{aligned}$$
- If $l \ge k$, $\alpha \in {\mathcal {A}}_{k}$ and $\beta \in {\mathcal {A}}_{l}$ then either $Q^{l}_{\beta } \subseteq Q^{k}_{\alpha }$ or $Q^{k}_{\alpha } \cap Q^{l}_{\beta } = \emptyset$;
- For every $l \in {\mathbb {Z}}$ and $\alpha \in {\mathcal {A}}_{l}$, there exists a point $z^{l}_{\alpha }$ with the property that
  $$\begin{aligned} B(z^{l}_{\alpha }, c_{0} \delta ^{l}) \subseteq Q^{l}_{\alpha } \subseteq B(z^{l}_{\alpha }, C_{0} \delta ^{l}). \end{aligned}$$

The point $z_{\alpha }^l$ can be seen as the centre of the cube $Q^{l}_{\alpha }$ and the side length is defined by $\ell (Q_{\alpha }^{l}) := \delta ^{l}$.

The below theorem asserts the existence of adjacent systems of dyadic cubes for a doubling metric space. For a proof of this result, refer to [31].

#### Theorem 2.2

[31, Thm. 4.1] Let $(M,d,\mu )$ be a doubling metric space. There exists $0< c_{0} \le C_{0} < \infty$, $\delta \in (0,1)$, finite constants $K = K(c_{0},C_{0},\delta )$ and $C = C(\delta )$, and a finite collection of dyadic systems $\mathscr {D}^{b}$ with parameters $(c_{0},C_{0},\delta )$, $b = 1,\cdots , K$ that satisfies the following property. For any ball $B = B(x,r) \subseteq M$, there exists $b \in \left\{ 1, \cdots , K \right\}$ and $Q \in \mathscr {D}^{b}$ such that

$$\begin{aligned} B \subseteq Q \quad and \quad \mathrm {diam}(Q) \le C r. \end{aligned}$$

From this point forward we fix a dyadic collection $\mathscr {D} :=\cup _{b = 1}^{K} \mathscr {D}^{b}$ as in the previous theorem. The following covering lemma will be useful in Sect. 5.

#### Lemma 2.3

[44, Lemma 2.2] Let $(M,d,\mu )$ be a doubling metric space with $\mathrm {diam} (M) = \infty$ and $\mathscr {D}$ a dyadic system with parameters $(c_0,C_0,\delta )$. Let $\alpha \ge 3/\delta$ and $E \subset M$ with $\mathrm {diam}(E) \in (0,\infty )$. There exists a partition $\mathscr {P} \subseteq \mathscr {D}$ of the space *M*, made with dyadic cubes, such that

$$\begin{aligned} E \subseteq \alpha Q \,,\quad \forall Q \in \mathscr {P}. \end{aligned}$$

Let *w* be a weight on *M*. The uncentered dyadic maximal function ${\mathcal {M}}^{\mathscr {D}}_{p,w}$ of exponent $p \in [1,\infty )$ is defined by

$$\begin{aligned} {\mathcal {M}}^{\mathscr {D}}_{p,w} f(x) :=\sup _{Q\in \mathscr {D}} \left( \frac{1}{w(Q)}\int _{Q} |f(y) |^{p} w(y) \, {\text {d}}{y} \right) ^{1/p} \mathbb {1}_Q(x), \end{aligned}$$

where the notation $\mathbb {1}_{E}$ is used to denote the characteristic function of a set $E \subset M$ and $w(E) :=\int _E w \, {\text {d}}{\mu }$. When $w \equiv 1$, ${\mathcal {M}}^{\mathscr {D}}_{p,w}$ will just be the usual dyadic maximal function of exponent *p* and the shorthand notation ${\mathcal {M}}^{\mathscr {D}}_{p} = {\mathcal {M}}^{\mathscr {D}}_{p,1}$ will be employed. Similarly, we will also use the notation ${\mathcal {M}}^{\mathscr {D}}_{w} = {\mathcal {M}}^{\mathscr {D}}_{1,w}$. It is known that ${\mathcal {M}}^{\mathscr {D}}_{p}$ is of weak-type (*p*, *p*) and strong (*q*, *q*) for all $q>p$, see [16]. Moreover, ${\mathcal {M}}^{\mathscr {D}}_w$ is bounded on $L^p(w)$ for all $p \in [1,\infty )$ with a constant independent of the weight,

2.1.1 $$\begin{aligned} \Vert {\mathcal {M}}^{\mathscr {D}}_w f \Vert _{L^p(w)} \le p' \Vert f \Vert _{L^p(w)}. \end{aligned}$$

### Off-Diagonal Estimates

In this section, we define three different notions of off-diagonal estimates that will be used throughout this article. For an extensive and detailed account of off-diagonal estimates for operator families, the reader is referred to [4]. Throughout this section, we will consider exponents $1 \le p_{0}< 2 < q_{0} \le \infty$.

#### Definition 2.4

(Off-diagonal estimates at scale $\sqrt{t}$) A family of operators $\{T_t\}_{t>0}$ is said to satisfy $(p_0,q_0)$ off-diagonal estimates at scale $\sqrt{t}$ if for any two balls $B_1,B_2$ of radius $\sqrt{t}$ we havewhere $\rho :[0,\infty ) \rightarrow (0,1]$ is a non-increasing function such that $\rho (0)=1$ and $\lim _{x\rightarrow \infty }|x|^a \rho (x)=0$ for some $a \ge 0$.

#### Remark 2.5

Some comments are in order.

- Examples of $\rho$ that we will use are the Gaussian function $\rho (x) = e^{-c|x|^2}$ and $\rho (x) = \langle x \rangle ^{-s}$, where $\langle x \rangle = (1 + |x |^2)^{1/2}$ is the Japanese bracket. For the Gaussian case, the positive constant *c* is not relevant and may change from line to line. See also comments after [4, Def. 2.1]. For our sparse domination, the choice $\rho (x)=\langle x\rangle ^{-2(\nu + 1)}$ suffices.
- Off-diagonal estimates at scale $\sqrt{t}$ are stable under composition. That is, if $T_{t}$ satisfies $(p_{1},p_{2})$ off-diagonal estimates at scale $\sqrt{t}$ and $S_{t}$ satisfies $(p_{2},p_{3})$ off-diagonal estimates at scale $\sqrt{t}$ then $S_{t} T_{t}$ will satisfy $(p_{1},p_{3})$ off-diagonal estimates at scale $\sqrt{t}$. It should be noted, however that the value of *c* or *s* in the above examples of $\rho$ may change for the composition.
- For $p_{0} \le p \le q \le q_{0}$, Hölder’s inequality implies that if an operator family satisfies $(p_{0},q_{0})$ off-diagonal estimates at scale $\sqrt{t}$ then it will also satisfy (*p*, *q*) estimates.
- Off-diagonal estimates for $p\le q$ do not imply $L^p-L^q$ boundness of $T_t$, see [4].

In order to apply off-diagonal estimates, we often need to decompose the support of a function *f* into finitely overlapping balls with radius to match the scale.

#### Definition 2.6

We say that a collection of balls ${\mathcal {B}}$ has finite overlap if there exists a finite constant $\Lambda _{{\mathcal {B}}}$ such that

$$\begin{aligned} \Vert \sum _{B \in {\mathcal {B}}} \mathbb {1}_B \Vert _{L^\infty } = \Lambda _{{\mathcal {B}}} . \end{aligned}$$

#### Remark 2.7

Let ${\mathcal {B}}$ be a collection of finite overlapping balls covering a set $\Omega$. Then

$$\begin{aligned} \sum _{B \in {\mathcal {B}}} \mu (B) = \int _{\Omega } \sum _{B \in {\mathcal {B}}} \mathbb {1}_B \, {\text {d}}{\mu } \le \Lambda _{{\mathcal {B}}} \; \mu (\Omega ) . \end{aligned}$$

#### Lemma 2.8

Let $\Omega \subset M$ be an open set, and let ${\mathcal {R}}$ be a family of finite overlapping balls, with the same radius, covering $\Omega$. If there exists $m \in {\mathbb {N}}$ such that $m R \supset \Omega$ for all $R \in {\mathcal {R}}$, then for any $f \in L^{p_0}(\Omega )$, $p_0 \ge 1$, we have2.2.1

#### Proof

For $p_0 >1$, Hölder’s inequality implies thatSince $m R \supset \Omega$ for all $R \in {\mathcal {R}}$, the doubling property implies that

$$\begin{aligned} \left( \sup _{R \in {\mathcal {R}}} \frac{|\Omega |}{\left|R \right|} \right) ^{\frac{1}{p_{0}}} \left( \# {\mathcal {R}} \right) ^{\frac{1}{p_{0}'}} \lesssim \sup _{R \in {\mathcal {R}}}\frac{|m R|}{\left|R \right|} \lesssim m^\nu . \end{aligned}$$

The case $p_{0} = 1$ is even simpler since it does not require the use of Hölder’s inequality nor an estimate on the cardinality $\# {\mathcal {R}}$. $\square$

#### Remark 2.9

If $T_s$ satisfies $(p_0,q_0)$ off-diagonal estimates at scale $\sqrt{s}$, then it satisfies2.2.2for balls *B*(*r*) of radius $r \ge \sqrt{s}$ and $B_{1}$ of radius $\sqrt{s}$.

#### Proof of (2.2.2)

It is enough to cover the larger ball *B*(*r*) with a collection ${\mathcal {B}}$ of smaller, finite overlapping balls of radius $\sqrt{s}$.We can use off-diagonal estimates at scale $\sqrt{s}$ to obtainThe estimate then follows from the fact that the supremum of $\rho (d(B,B_1)/\sqrt{s})$ over $B \in {\mathcal {B}}$ is at most $\rho \big (d(B(r),B_1)/\sqrt{s}\big )$. $\square$

We denote the semigroup by $P_t :=e^{-tL}$. This is used as an approximation of the identity at scale $\sqrt{t}$, since for any $p \in (p_0,q_0)$ we have

$$\begin{aligned} \lim _{t \rightarrow 0} \Vert f - e^{-tL}f \Vert _{L^p} = 0 \quad \text { and } \quad \lim _{ t \rightarrow \infty } \Vert e^{-tL} f \Vert _{L^p} = 0 . \end{aligned}$$

For $N > 0$, we also consider the family of operators $Q_t^{(N)} :=c_{N}^{-1}(tL)^{N} e^{-tL}$ with $c_{N} = \int ^{\infty }_{0} s^{N} e^{-s}\frac{ \, {\text {d}}{s}}{s}$. These operators will satisfy an adapted Calderón reproducing formula for functions $f \in L^p$ with $p \in (p_0,q_0)$, namely

$$\begin{aligned} f = \int _0^\infty Q_t^{(N)} f \frac{ \, {\text {d}}{t}}{t} . \end{aligned}$$

Also define

$$\begin{aligned} P_{t}^{(N)} := \int ^{\infty }_{1} Q_{st}^{(N)} \, \frac{ \, {\text {d}}{s}}{s}. \end{aligned}$$

Then $P_t^{(N)}$ is related to the operator $Q_{t}^{(N)}$ through $t\partial _t P_t^{(N)} = - Q_t^{(N)}$. We also have that as $L^{p}$-bounded operators,

$$\begin{aligned} P_t^{(N)} = \mathrm {Id} + \int _0^t Q_s^{(N)} \frac{ \, {\text {d}}{s}}{s} . \end{aligned}$$

#### Remark 2.10

It is known that for any integer $N \in {\mathbb {N}} \setminus \left\{ 0 \right\}$ the operators $P_t^{(N)}$ and $Q_t^{(N)}$ satisfy (*p*, *p*) off-diagonal estimates at scale $\sqrt{t}$ for all $t>0$ and all $p \in [p_0,q_0]$ with $p < \infty$ (see the arguments in [29, Prop 3.1], for instance).

#### Definition 2.11

(Off-diagonal estimates at all scales) A family of operators $\{T_t\}_{t>0}$ is said to satisfy $(p_0,q_0)$ off-diagonal estimates at all scales if for all balls $B_{1}, \, B_{2}$ of radius $r_1,r_2$ we have

$$\begin{aligned} \left\Vert T_{t} \right\Vert _{L^{p_{0}}(B_{1}) \rightarrow L^{q_{0}}(B_{2})} \lesssim \big |B_{1,\sqrt{t}}\big |^{-\frac{1}{p_{0}}} \big |B_{2,\sqrt{t}}\big |^{\frac{1}{q_{0}}} \rho \left( \frac{d(B_{1},B_{2})}{\sqrt{t}} \right) , \end{aligned}$$

where $B_{i,\sqrt{t}} := (\sqrt{t}/r_i) B_{i}$ for $i = 1, \, 2$ and $\rho :[0,\infty ) \rightarrow (0,1]$ is a non-increasing function such that $\rho (0)=1$ and $\lim _{x\rightarrow \infty }|x|^a \rho (x)=0$ for some $a \ge 0$.

It is trivial to see that off-diagonal estimates at all scales implies off-diagonal estimates at scale $\sqrt{t}$. This stronger condition is used in our cancellation hypothesis, Assumption 1.2(c).

Let $\psi : (0,\infty ) \rightarrow (0,\infty )$ be a non-decreasing function. A space of homogeneous type $(M,\mu )$ is said to be of $\psi$-growth if

$$\begin{aligned} \left|B(x,r) \right| = \mu (B(x,r)) \simeq \psi (r) \end{aligned}$$

uniformly for all $x \in M$ and $r > 0$. Notice that this condition is stronger than (1.0.4). For spaces of $\psi$-growth, one encounters another notion of off-diagonal estimate. These types of estimates are studied in [4].

#### Definition 2.12

(Full off-diagonal estimates) Suppose that $(M,\mu )$ is of $\psi$-growth. A family of operators $\{T_t\}_{t>0}$ is said to satisfy $(p_0,q_0)$ full off-diagonal estimates if for all closed sets $E, \, F$ we have

$$\begin{aligned} \left\Vert T_{t} \right\Vert _{L^{p_{0}}(E) \rightarrow L^{q_{0}}(F)} \lesssim \psi (\sqrt{t})^{\frac{1}{q_{0}} - \frac{1}{p_{0}}}\rho \left( \frac{d(E,F)}{\sqrt{t}} \right) , \end{aligned}$$

where $\rho :[0,\infty ) \rightarrow (0,1]$ is a non-increasing function such that $\rho (0)=1$ and $\lim _{x\rightarrow \infty }|x|^a \rho (x)=0$ for some $a \ge 0$.

#### Remark 2.13

It is not difficult to show that for spaces of $\psi$-growth, the three different notions of off-diagonal estimates, Definitions 2.4, 2.11 and 2.12, are all equivalent for a particular choice of $\rho$.

### Weight Classes

We recall some basic properties of the Muckenhoupt and reverse Hölder weight classes as defined in the introduction. Refer to [33] for further information.

#### Lemma 2.14

The following properties of the weight classes $A_{p}$ and $RH_{q}$ are true.

(i) For $p \in (1,\infty )$, a weight *w* will be contained in the class $A_{p}$ if and only if $w^{1 - p'} \in A_{p'}$. Moreover,
  $$\begin{aligned} \big [w^{1-p'}\big ]_{A_{p'}} = \big [w\big ]^{p' - 1}_{A_{p}}. \end{aligned}$$
(ii) For $q \in [1,\infty ]$ and $s \in [1,\infty )$, a weight *w* will be contained in $A_{q} \cap RH_{s}$ if and only if $w^{s} \in A_{s(q - 1) + 1}$. Moreover,
  $$\begin{aligned} \max \{\left[ w \right] ^{s}_{A_{q}} , \left[ w \right] ^{s}_{RH_{s}}\} \le \left[ w^{s} \right] _{A_{s(q - 1) + 1}} \le \left[ w \right] ^{s}_{A_{q}} \left[ w \right] ^{s}_{RH_{s}}. \end{aligned}$$

For $1\le p_{0}< 2 < q_{0} \le \infty$ and $p \in (p_{0},q_{0})$ define

$$\begin{aligned} \phi (p) :=\left( \frac{q_{0}}{p} \right) ' \left( \frac{p}{p_{0}} - 1 \right) + 1. \end{aligned}$$

The dependence of $\phi$ on $p_{0}$ and $q_{0}$ will be kept implicit. From the previous lemma, we get that a weight *w* will be contained in the class $A_{\frac{p}{p_{0}}} \cap RH_{(\frac{q_{0}}{p})'}$ if and only if $w^{(\frac{q_{0}}{p})'}$ is contained in $A_{\phi (p)}$ and it will be true that

2.3.1 $$\begin{aligned} \left[ w^{(\frac{q_{0}}{p})'} \right] _{A_{\phi (p)}} \le \left( \left[ w \right] _{A_{\frac{p}{p_{0}}}} \left[ w \right] _{RH_{(\frac{q_{0}}{p})'}} \right) ^{(\frac{q_{0}}{p})'}. \end{aligned}$$

In the article [3], the authors P. Auscher and J. M. Martell proved a restricted range extrapolation result that allowed one to obtain $L^{p}(w)$-boundedness for the full range of $p \in (p_{0},q_{0})$ and $w \in A_{\frac{p}{p_{0}}} \cap RH_{(\frac{q_{0}}{p})'}$ directly from the $L^{q}(w)$-boundedness for all $w \in A_{\frac{q}{p_{0}}} \cap RH_{(\frac{q_{0}}{q})'}$ of a single index $q \in (p_{0},q_{0})$. In their result, they do not state the dependence of the bound on the weight characteristic $\left[ w^{(\frac{q_{0}}{p})'} \right] _{A_{\phi (p)}}$. However, a quantitative version of the extrapolation theorem by Auscher and Martell can be obtained through [46, Thm. 2.2] in the scalar case ($m=1$), as their weight characteristic $[w]_{p,(r,s)}$ coincides (up to a power) with $[w^{(q_0/p)'}]_{A_{\phi (p)}}$ when $r=p_0$ and $s=q_0$.

Here we recall this result using the notation of [3, Thm. 4.9] and the weight characteristic introduced earlier. As in [50], ${\mathcal {F}}$ denotes a family of ordered pairs of non-negative, measurable functions (*f*, *g*).

#### Theorem 2.15

(Sharp Restricted Range Extrapolation) Let $0< p_{0} < q_{0} \le \infty$. Suppose that there exists *q* with $p_{0} \le q < q_{0}$ such that for $(f,g) \in {\mathcal {F}}$,

$$\begin{aligned} \left\Vert f \right\Vert _{L^{q}(w)} \le C \left[ w^{(\frac{q_{0}}{q})'} \right] _{A_{\phi (q)}}^{\alpha } \left\Vert g \right\Vert _{L^{q}(w)} \quad \text { for all } w\in A_{\frac{q}{p_{0}}} \cap RH_{(\frac{q_{0}}{q})'}, \end{aligned}$$

for some $\alpha > 0$ and $C > 0$ independent of the weight. Then, for all $p_{0}< p < q_{0}$ and $(f,g) \in {\mathcal {F}}$ we have

$$\begin{aligned} \left\Vert f \right\Vert _{L^{p}(w)} \le C' \left[ w^{(\frac{q_{0}}{p})'} \right] _{A_{\phi (p)}}^{\beta (p,q)\cdot \alpha } \left\Vert g \right\Vert _{L^{p}(w)} \quad \text { for all } w \in A_{\frac{p}{p_{0}}} \cap RH_{(\frac{q_{0}}{p})'}, \end{aligned}$$

where $\beta (p,q) :=\max \left( 1, \frac{(q_{0} - p)(q - p_{0})}{(q_{0} - q)(p - p_{0})} \right)$ and $C' > 0$ is independent of the weight.

## Applications

In this section, we consider two distinct applications of our quadratic sparse domination result and Corollary 1.9. For the first application, weighted estimates for square functions associated with divergence form elliptic operators will be proved. For the particular case of the Laplacian operator $\Delta$, this will allow us to recover some estimates from [13]. The second example that we will look at are square functions associated with the Laplace–Beltrami operator on a Riemannian manifold.

### Elliptic Operators

Fix $n \in {\mathbb {N}}\setminus \left\{ 0 \right\}$ and consider the Euclidean space ${\mathbb {R}}^n$ equipped with the Lebesgue measure. This is a space of $\psi$-growth, so all definitions of off-diagonal estimates are equivalent, see Remark 2.13.

Let *A* be an $n \times n$ matrix-valued function on ${\mathbb {R}}^{n}$ that is bounded and elliptic in the sense that

$$\begin{aligned} \mathrm {Re} \langle A(x) \xi , \xi \rangle _{{\mathbb {C}}^{n}} \ge \lambda \left|\xi \right|^{2}, \end{aligned}$$

for some $\lambda > 0$, for all $\xi , \, x \in {\mathbb {R}}^{n}$. Consider the divergence form elliptic operator

$$\begin{aligned} L = - \mathrm {div} A \nabla , \end{aligned}$$

defined through its corresponding sesquilinear form as a densely defined and maximally accretive operator on $L^{2}({\mathbb {R}}^{n})$. The operator *L* generates an analytic semigroup $\left\{ e^{-z L} \right\} _{z \in \Sigma _{\pi /2 - \theta }}$, where

$$\begin{aligned} \theta :=\sup \left\{ \left|\mathrm {arg} \langle L f, f \rangle \right| : f \in {\mathcal {D}}_{2}(L) \right\} . \end{aligned}$$

Let $g_{L}$ and $G_{L}$ denote the square function operators associated with *L* defined by

$$\begin{aligned} g_{L}f :=\left( \int ^{\infty }_{0} \left|t L \,e^{-t L} f \right|^{2} \, \frac{ \, {\text {d}}{t}}{t} \right) ^{1/2} \quad \text {and} \quad G_{L}f :=\left( \int ^{\infty }_{0} \left|\sqrt{t} \nabla e^{-t L}f \right|^{2} \, \frac{ \, {\text {d}}{t}}{t} \right) ^{1/2}. \end{aligned}$$

In the articles [4] and [2], off-diagonal estimates for the constituent operators of $g_{L}$ and $G_L$ were studied in great detail. The below proposition outlines some properties of such off-diagonal estimates that will be required in order to apply Corollary 1.9 to these two square functions.

#### Proposition 3.1

[2, Prop. 3.3] For $m \in {\mathbb {N}}$ and $0< \mu < \pi /2 - \theta$, there exists maximal intervals ${\mathcal {J}}^{m}(L)$ and ${\mathcal {K}}^{m}(L)$ in $[1,\infty ]$ satisfying the below properties.

- If $p_{0}, \, q_{0} \in {\mathcal {J}}^{m}(L)$ with $p_{0}\le q_{0}$ then $\left\{ (z L)^{m} e^{-z L} \right\} _{z \in \Sigma _{\mu }}$ satisfies $(p_{0},q_{0})$ full off-diagonal estimates.
- If $p_{0}, \, q_{0} \in {\mathcal {K}}^{m}(L)$ with $p_{0}\le q_{0}$ then $\left\{ \sqrt{z} \nabla (z L)^{m} e^{-z L} \right\} _{z \in \Sigma _{\mu }}$ satisfies $(p_{0},q_{0})$ full off-diagonal estimates.
- The interiors $\mathrm {int} \, {\mathcal {J}}^{m}(L)$ and $\mathrm {int} \, {\mathcal {K}}^{m}(L)$ are independent of *m*.
- The inclusion ${\mathcal {K}}^{m}(L) \subseteq {\mathcal {J}}^{m}(L)$ is satisfied.
- The point $p=2$ is contained in ${\mathcal {K}}^{m}(L)$.

#### Remark 3.2

Observe that for any $m \ge 1$, ${\mathcal {J}}^{1}(L) \subset {\mathcal {J}}^{m}(L)$. To see this, let $p_{0}, q_{0} \in {\mathcal {J}}^{1}(L)$ with $p_{0} \le q_{0}$. Then $(t L)e^{-t L/m}$ must satisfy both $(p_{0},q_{0})$ and $(q_{0},q_{0})$ off-diagonal estimates. This fact, when combined with the decomposition

$$\begin{aligned} (t L)^{m} e^{-t L} = (t L) e^{-t L/m} \cdots (t L) e^{-t L / m} \end{aligned}$$

and the property that full off-diagonal estimates are stable under composition (c.f. [4, Thm. 2.3 (b)]) then implies that $p_{0}, \, q_{0}\in {\mathcal {J}}^{m}(L)$.

It is also not difficult to see that ${\mathcal {J}}^{0}(L) \subset {\mathcal {J}}^{1}(L)$. Indeed, consider the expression

$$\begin{aligned} t L e^{-t L} = e^{-\frac{t}{3}L} \cdot (t L) e^{-\frac{t}{3} L} \cdot e^{-\frac{t}{3} L}. \end{aligned}$$

For $p_{0}, \, q_{0} \in {\mathcal {J}}^{0}(L)$ with $p_{0}< 2 < q_{0}$, Proposition 3.1 tells us that the operator $e^{-\frac{t}{3}L}$ will satisfy both $(p_{0},2)$ and $(2,q_{0})$ full off-diagonal estimates. It is also well known that $t L e^{-\frac{t}{3} L}$ satisfies (2, 2) full off-diagonal estimates. The stability of full off-diagonal estimates under composition then implies that $t L e^{-t L}$ satisfies $(p_{0},q_{0})$ full off-diagonal estimates.

Applying Corollary 1.9 to the operators *L* and $g_{L}$ will produce the following weighted result.

#### Proposition 3.3

Let $p_{0}, \, q_{0} \in {\mathcal {J}}^{0}(L)$ with $p_{0}< 2 < q_{0}$. Then, for any $p \in (p_{0}, q_{0})$ and $w \in A_{\frac{p}{p_{0}}} \cap RH_{(\frac{q_{0}}{p})'}$,

$$\begin{aligned} \left\Vert g_{L} \right\Vert _{L^{p}(w)} \lesssim \left( \left[ w \right] _{A_{\frac{p}{p_{0}}}} \cdot \left[ w \right] _{RH_{(\frac{q_{0}}{p})'}} \right) ^{\gamma (p)}, \end{aligned}$$

where $\gamma (p)$ is as defined in Corollary 1.9.

#### Proof

To prove the proposition, it is sufficient to check that the hypotheses of Corollary 1.9, namely Assumptions 1.1 and 1.2, are valid for the operators *L* and $g_{L}$ and the indices $p_{0}, \, q_{0}$. Assumption 1.1 is clearly valid since the definition of ${\mathcal {J}}^{0}(L)$ implies that the semigroup $e^{-t L}$ will satisfy $(p_{0},q_{0})$ full off-diagonal estimates.

It remains to prove the validity of Assumption 1.2. Part (a), the $L^{2}$-boundedness of $g_{L}$, follows from the fact that *L* possesses a bounded holomorphic functional calculus on $L^{2}$. Assumption 1.2(b), the off-diagonal estimates of the operator family $t L e^{-t L}$ is given by Remark 3.2. Assumption 1.2(c) follows on observing that

$$\begin{aligned} {\mathcal {Q}}_{s} (t L)^{N} e^{-t L}&= s L e^{-s L} (tL)^{N} e^{-t L} \\&= \frac{s t^{N}}{(s + t)^{N + 1}} ((s + t) L)^{N + 1} e^{- (s + t)L} \end{aligned}$$

and that since $p_{0}, \, q_{0} \in {\mathcal {J}}^{0}(L)$ the operator family $\Theta _{r}^{(N)} = (r L)^{N + 1} e^{-r L}$ will possess $(p_{0},q_{0})$ full off-diagonal bounds for any $N \ge N_{0} = 0$ by Remark 3.2. Finally, for Assumption 1.2(d), in the proof of [2, Thm. 7.2 (a)] it was shown that for any ball *B*(*x*, *r*) we have3.1.1for some sequence of numbers $c(j) > 0$ that satisfies $\sum _{j \ge 1} c(j) \lesssim 1$. It should be noted that this argument was written for the square function with constituent operators $(t L)^{\frac{1}{2}} e^{-t L}$, but it applies equally well to our choice of square function. This clearly implies thatand thus, Assumption 1.2(d) is valid. $\square$

Similarly, Corollary 1.9 can be applied to the square function $G_{L}$.

#### Proposition 3.4

Let $p_{0}, \, q_{0} \in {\mathcal {K}}^{0}(L)$ with $p_{0}< 2 < q_{0}$. Then, for any $p \in (p_{0}, q_{0})$ and $w \in A_{\frac{p}{p_{0}}} \cap RH_{(\frac{q_{0}}{p})'}$,

$$\begin{aligned} \left\Vert G_{L} \right\Vert _{L^{p}(w)} \lesssim \left( \left[ w \right] _{A_{\frac{p}{p_{0}}}} \cdot \left[ w \right] _{RH_{(\frac{q_{0}}{p})'}} \right) ^{\gamma (p)}. \end{aligned}$$

#### Proof

In order to apply Corollary 1.9, it is sufficient to show that $G_{L}$ satisfies Assumptions 1.1 and 1.2. Assumption 1.1 is implied by $p_{0}, \, q_{0} \in {\mathcal {K}}^{0}(L) \subset {\mathcal {J}}^{0}(L)$.

Let us now demonstrate the validity of Assumption 1.2. The $L^{2}$-boundedness of $G_{L}$, Assumption 1.2(a), follows from the ellipticity condition of *A* and a straightforward integration by parts argument that can be found in [1, pg. 74]. Assumption 1.2(b) is implied by the condition $p_{0}, \, q_{0} \in {\mathcal {K}}^{0}(L)$. For Assumption 1.2(c), notice that

$$\begin{aligned}\begin{aligned} {\mathcal {Q}}_{s} Q_{t}^{(N)}&= \sqrt{s} \nabla e^{-s L} (t L)^{N} e^{-t L} \\&= \frac{s^{\frac{1}{2}} t^{N}}{(s + t)^{N + \frac{1}{2}}} \sqrt{s + t} \nabla \left( (s + t) L \right) ^{N} e^{-(s + t)L} \\&=: \frac{s^{\frac{1}{2}} t^{N}}{(s + t)^{N + \frac{1}{2}}} \Theta ^{(N)}_{s + t}. \end{aligned}\end{aligned}$$

Also observe that

$$\begin{aligned} \Theta ^{(N)}_{r} = \sqrt{r} \nabla e^{- r L / 2} (r L)^{N} e^{-r L/2}. \end{aligned}$$

As $p_{0}, \, q_{0} \in {\mathcal {K}}^{0}(L)$, Proposition 3.1 tells us that operator family $\sqrt{r} \nabla e^{-r L/2}$ will satisfy $(2,q_{0})$ full off-diagonal estimates. Similarly, since ${\mathcal {K}}^{0}(L) \subset {\mathcal {J}}^{N}(L)$ for any $N \ge N_{0} = 0$, the family $(r L)^{N} e^{-r L/2}$ satisfies $(p_{0},2)$ full off-diagonal bounds. It then follows from the stability of full off-diagonal bounds under composition that the family $\Theta _{r}^{(N)}$ will satisfy $(p_{0},q_{0})$ full off-diagonal bounds. This proves that Assumption 1.2(c) is satisfied.

Finally, for Assumption 1.2(d), in the proof of [2, Thm. 7.2 (b)] it was proved that for any ball *B*(*x*, *r*) we havefor some sequence of numbers $d(j) > 0$ that satisfies $\sum _{j \ge 1} d(j) \lesssim 1$. This clearly implies that3.1.2and thus, Assumption 1.2(d) is valid. $\square$

#### Remark 3.5

If *A* is real valued, then it is known that ${\mathcal {J}}^{0}(L) = [1,\infty ]$ (c.f. [2]). Proposition 3.3 will then imply that

$$\begin{aligned} \left\Vert g_{L} \right\Vert _{L^{p}(w)} \lesssim \left[ w \right] ^{\max \left( \frac{1}{p - 1}, \frac{1}{2} \right) }_{A_{p}} \end{aligned}$$

for all $w \in A_{p}$. When *A* has smooth coefficients, this result was proved by Bui and Duong in [13].

In the same work, the authors showed that square functions associated with $\sqrt{L}$ are dominated by the corresponding one associated with *L* [13, Thm. 1.4].

In particular, our bounds for $g_L$ in Proposition 3.3 implies the same bound for the square function $g_{\sqrt{L}}$. If, in addition to being real valued, *A* is also smooth then it is known that ${\mathcal {K}}^{0}(L) = [1,\infty ]$. Proposition 3.4 then implies that

$$\begin{aligned} \left\| G_{L} \right\| _{L^{p}(w)} \lesssim [w]_{A_{p}}^{\max \left( \frac{1}{p-1},\frac{1}{2}\right) }, \end{aligned}$$

which reproduces a result in [13].

#### Remark 3.6

For $A = I$ we have $L = \Delta$ and it is then known that ${\mathcal {J}}^{0}(L) = {\mathcal {K}}^{0}(L) = [1,\infty ]$. We can then take $p_{0} = 1$ and $q_{0} = \infty$ in Propositions 3.3 and 3.4. This will produce the weighted estimates

$$\begin{aligned}\begin{aligned} \left\Vert g_{\Delta } \right\Vert _{L^{p}(w)}, \; \left\Vert G_{\Delta } \right\Vert _{L^{p}(w)}&\lesssim \left( \left[ w \right] _{A_{p}} \left[ w \right] _{RH_{1}} \right) ^{\max \left( \frac{1}{p - 1},\frac{1}{2} \right) } = \left[ w \right] ^{\max \left( \frac{1}{p - 1}, \frac{1}{2} \right) }_{A_{p}} \end{aligned}\end{aligned}$$

for all $w \in A_{p} \cap RH_{1} = A_{p}$. For both square functions, it is known that these estimates are optimal in the sense that they will not hold for an exponent of $\left[ w \right] _{A_{p}}$ any smaller than the above exponent. This provides a new proof of weighted boundedness of the standard square functions associated with $\Delta$ with optimal dependence on the constant $\left[ w \right] _{A_{p}}$.

### Laplace–Beltrami

Let *M* be a complete, connected, non-compact Riemannian manifold. It will be assumed that the Riemannian measure $\mu$ satisfies the volume doubling property. In addition, it will also be assumed that there exists a function $\psi :(0,\infty ) \rightarrow (0,\infty )$ for which

$$\begin{aligned} \left|B(x,r) \right| = \mu (B(x,r)) \simeq \psi (r) \end{aligned}$$

uniformly for all $x \in M$ and $r > 0$. That is, the manifold is of $\psi$-growth. Enforcing this stronger growth condition will allow us to interchange our different notions of off-diagonal estimates (c.f. Remark 2.13).

Consider the Laplace–Beltrami operator $\Delta$ defined as an unbounded operator on $L^{2}(M,\mu )$ through the integration by parts formula

$$\begin{aligned} \langle \Delta f, f \rangle = \left\Vert \left|\nabla f \right| \right\Vert ^{2}_{2} \end{aligned}$$

for $f \in C^{\infty }_{0}(M)$, where $\nabla$ is the Riemannian gradient. The positivity of $\Delta$ implies that it will generate an analytic semigroup $e^{-t \Delta }$ on $L^{2}(M,\mu )$.

Recall that the heat kernel $k_{t}(x,y)$ of $\Delta$ is said to satisfy Gaussian upper bounds if there exists $c > 0$ such that

$$\begin{aligned} k_{t}(x,y) \lesssim \frac{1}{\left|B(x,\sqrt{t}) \right|} e^{-c \frac{d^{2}(x,y)}{t}} \end{aligned}$$

for all $x, \, y \in M$ and $t > 0$. This is a very common assumption that is imposed when considering the boundedness of singular operators on Riemannian manifolds. For further information refer to [6, 19] or [5]. Consider the square function $g_{\Delta }$ defined through

$$\begin{aligned} g_{\Delta } f :=\left( \int ^{\infty }_{0} \left|t \Delta e^{-t\Delta }f \right|^2 \frac{ \, {\text {d}}{t}}{t}\right) ^{1/2}. \end{aligned}$$

The boundedness for square functions of this form on unweighted $L^{p}(M)$ with $1< p < \infty$ is known to hold in the general symmetric Markov semigroup setting [49, pg. 111]. Let us consider the weighted case on the full range of $p \in (1,\infty )$.

#### Proposition 3.7

Suppose that the heat kernel for *M* satisfies Gaussian upper bounds. Then, for any $p \in (1,\infty )$ and $w \in A_{p}$,

$$\begin{aligned} \left\Vert g_{\Delta } \right\Vert _{L^{p}(w)} \lesssim \left[ w \right] _{A_{p}}^{\max \left( \frac{1}{2}, \frac{1}{p - 1} \right) }. \end{aligned}$$

#### Proof

This result will follow from Corollary 1.9 provided that Assumptions 1.1 and 1.2 are verified to hold with $p_{0} = 1$ and $q_{0} = \infty$.

For Assumption 1.1, it is known that the heat kernel satisfying Gaussian upper bounds is equivalent to the semigroup $e^{-t\Delta }$ satisfying $(1,\infty )$ full off-diagonal estimates. For proof, the reader is referred to [4, Prop. 2.2] and [4, Prop. 3.3]. Thus, Assumption 1.1 will be valid.

For Assumption 1.2(a), the $L^{2}$-boundedness of $g_{\Delta }$ follows from the bounded holomorphic functional calculus of $\Delta$ on $L^{2}$. For Assumption 1.2(b), notice that

$$\begin{aligned} t \Delta e^{-t \Delta } = e^{-\frac{t}{3} \Delta } \cdot t\Delta e^{-\frac{t}{3} \Delta } \cdot e^{-\frac{t}{3} \Delta }. \end{aligned}$$

Observe that since the semigroup $e^{-t \Delta }$ satisfies $(1,\infty )$ full off-diagonal estimates, $e^{-t \Delta }$ will satisfy both (1, 2) and $(2,\infty )$ full off-diagonal bounds. At the same time, $t\Delta e^{-t\Delta }$ is well known to satisfy (2, 2) full off-diagonal bounds (c.f. [6, pg. 930] and [22, Lemma 7]). It then follows from the stability of full off-diagonal bounds under composition ( [4, Thm. 2.3 (b)]) that $t \Delta e^{-t\Delta }$ satisfies $(1,\infty )$ full off-diagonal bounds. This proves that Assumption 1.2(b) is satisfied.

Assumption 1.2(c) follows from the expression

$$\begin{aligned} {\mathcal {Q}}_{s} (t\Delta )^{N} e^{-t \Delta } = \frac{s t^{N}}{(s + t)^{N + 1}} \left[ (s + t) \Delta \right] ^{N + 1} e^{-(s + t)\Delta } \end{aligned}$$

and the fact that the operator family $\{(r \Delta )^{N + 1}e^{- r \Delta }\}_{r>0}$ satisfies $(1,\infty )$ full off-diagonal bounds by an argument similar to that of Remark 3.2.

Finally, the validity of Assumption 1.2(d) can be proved in an identical manner to the argument used to obtain (3.1.1). This argument can be found in [2, §7] on pages 729–730. This argument in the elliptic setting follows from a combination of the off-diagonal estimates of the constituent operators, the fact that the constituent operators are expressible in terms of the semigroup and a variation of the Marcinkiewicz–Zygmund theorem [27]*Thm. 5.5.1. All three of these components will hold for our square function in this Riemannian manifold setting, and thus, the argument will be valid. $\square$

Next, we will apply our sparse result to the square function

$$\begin{aligned} G_{\Delta }f :=\left( \int ^{\infty }_{0} \left|\sqrt{t} \nabla e^{-t\Delta } f \right|^{2} \frac{ \, {\text {d}}{t}}{t}\right) ^{1/2}. \end{aligned}$$

Define

$$\begin{aligned} q_{+} :=\sup \left\{ p \in (1,\infty ) : \left\Vert \left| \nabla \Delta ^{-\frac{1}{2}} f \right| \right\Vert _{p} \lesssim \left\Vert f \right\Vert _{p} \right\} . \end{aligned}$$

The weighted boundedness of the Riesz transforms operator $\nabla \Delta ^{-\frac{1}{2}}$ on $L^{p}(M,w \, {\text {d}}{\mu })$ was considered for $p \in (1,q_{+})$ in [5]. Owing to the strong connection between the Riesz transforms and the square function $G_{\Delta }$, the range $(1,q_{+})$ will also be a natural interval over which to consider the boundedness of $G_{\Delta }$. From the definition of $q_{+}$ and the $L^{2}$-boundedness of $\nabla \Delta ^{-\frac{1}{2}}$, it is clear that $q_{+} \ge 2$. In the below proposition we assume this inequality to be strict.

#### Proposition 3.8

Assume that the heat kernel of *M* satisfies Gaussian upper bounds and that $q_{+} > 2$. Let $2< q_{0} < q_{+}$ and $p \in [1,q_{0})$. Then for any $w \in A_{p} \cap RH_{(\frac{q_{0}}{p})'}$,

$$\begin{aligned} \left\Vert G_{\Delta } \right\Vert _{L^{p}(w)} \lesssim \left( \left[ w \right] _{A_{p}} \cdot \left[ w \right] _{RH_{(\frac{q_{0}}{p})'}} \right) ^{\gamma (p)}. \end{aligned}$$

#### Proof

Once again, let us apply Corollary 1.9. Assumption 1.1 will be true for the same reason as in Proposition 3.7. Assumption 1.2(a) is well known and can be obtained by combining the $L^{2}$-boundedness of $\nabla \Delta ^{-\frac{1}{2}}$ together with the bounded holomorphic functional calculus of $\Delta$ on $L^{2}$.

Let us show that the family of operators ${\mathcal {Q}}_{t} = \sqrt{t} \nabla e^{-t \Delta }$ satisfies $(1,q_{0})$ off-diagonal estimates at scale $\sqrt{t}$ with $\rho (x) = \exp (-cx^2)$, for some $c>0$. Fix balls $B_{1}, \, B_{2} \subset M$ of radius $\sqrt{t}$. From the argument in the proof of [6, Prop 1.10],

$$\begin{aligned} \left( \int _{M} \left|\nabla _{x} k_{t}(x,y) \right|^{q_{0}} e^{c \frac{d^{2}(x,y)}{t}} \, {\text {d}}{\mu }(x) \right) ^{\frac{1}{q_{0}}} \lesssim \frac{1}{\sqrt{t} \left|B(y,\sqrt{t}) \right|^{1 - \frac{1}{q_{0}}}} \end{aligned}$$

for all $t > 0$ and $y \in M$, where $c > 0$ is dependent on $q_{0}$. This immediately implies thatwhere the last line follows from the uniform $\psi$-growth condition imposed upon our manifold. For *f* supported in $B_{1}$, Minkowski’s inequality followed by the previous estimate producesLet us now prove that Assumption 1.2(c) is valid. Observe that

$$\begin{aligned} {\mathcal {Q}}_{s} (t \Delta )^{N} e^{- t \Delta }= & {} \frac{s^{\frac{1}{2}} t^{N}}{(s + t)^{N + \frac{1}{2}}} \sqrt{s + t} \nabla e^{-\frac{s + t}{2} \Delta } \left[ (s + t) \Delta \right] ^{N} e^{-\frac{s + t}{2} \Delta } \\=: & {} \frac{s^{\frac{1}{2}} t^{N}}{(s + t)^{N + \frac{1}{2}}} \Theta ^{(N)}_{s + t}. \end{aligned}$$

Observe that the operator family $\{(r \Delta )^{N} e^{-r \Delta }\}_{r>0}$ satisfies $(1,\infty )$ full off-diagonal estimates. Recall that for spaces of $\psi$-growth the three different forms of off-diagonal estimates, Definitions 2.4, 2.11 and 2.12, are all equivalent. This, when combined with Hölder’s inequality, implies that this operator family satisfies (1, 2) off-diagonal estimates at scale $\sqrt{r}$. Similarly, the family $\{\sqrt{r}\nabla e^{-r \Delta }\}_{r>0}$ satisfies $(2,q_{0})$ off-diagonal estimates at scale $\sqrt{r}$. The stability of off-diagonal estimates under composition then implies that the operator family $\Theta _{r}$ satisfies $(1,q_{0})$ at scale $\sqrt{r}$, which implies $(1,q_{0})$ off-diagonal estimates at all scales. This proves Assumption 1.2(c).

Finally, the validity of Assumption 1.2 (d) can be proved in an identical manner to the argument used to obtain (3.1.2). This argument can be found in [2, §7] on page 732. This argument in the elliptic setting follows from a combination of the off-diagonal estimates of the constituent operators, the fact that the constituent operators are expressible in terms of the semigroup and a variation of the Marcinkiewicz–Zygmund theorem [27]*Thm. 5.5.1. All three of these components will hold for our square function in this Riemannian manifold setting and thus the argument will be valid. $\square$

## Boundedness of the Maximal Function

Throughout this section, fix $p_{0}, \, q_{0} \in [1,\infty ]$, $N_{0} \in {\mathbb {N}}$ and operators *L* and *S* satisfying Assumptions 1.1 and 1.2 for such a choice of $p_{0}, \, q_{0}$. For a ball *B* we denote by *r*(*B*) its radius. Define the following maximal operator associated with the square function,In this section, our aim is to prove the following boundedness result for $S^{*}$.

### Theorem 4.1

The maximal function $S^{*}$ is bounded on $L^{2}$ and weak-type $(p_{0},p_{0})$.

The boundedness of this maximal function constitutes an important part of our sparse domination argument. The reliance of our argument on an associated maximal function is a well-known method for obtaining sparse bounds and finds its origins in the work of Lacey [35]. It was later streamlined by Lerner [42]. Quite often, the issue of proving sparse domination for a particular operator can be reduced to determining an appropriate associated maximal operator, proving its (weak) boundedness and then applying a stopping time argument that utilises this boundedness.

### A Pointwise Estimate

In order to prove the boundedness of the operator $S^{*}$ we will require a couple of preliminary lemmas. Given a ball *B*, we define the average of a function *f* over the annulus $S_k(B) :=2^{k+1} B \setminus 2^k B$ for $k\in {\mathbb {N}}$ as the integral over $S_k(B)$ normalised by $|2^k B|$.

Recall that $A_0$ is a positive number defined in Assumption 1.2 (c).

#### Lemma 4.2

For any $0< s< r^{2} < t$ and $N \in {\mathbb {N}}$,for any ball *B* of radius *r* and ${\widetilde{B}} := \frac{\sqrt{t}}{r}B$.

#### Proof

Fix *B* a ball of radius *r*. For $j \ge 0$, let ${\mathcal {R}}_{j}$ denote a collection of finite overlapping balls of radius $\sqrt{t}$ that is a cover for the set $S_{j}({\widetilde{B}})$. Then, Assumption 1.2 (c) together with the triangle inequality produces4.1.1On utilising the doubling property of our metric space and subsequently $s + t \simeq t$,

4.1.2 $$\begin{aligned} \begin{aligned} \left|B_{\sqrt{s + t}} \right|&\lesssim \left( \frac{\sqrt{s + t}}{r} \right) ^{\nu } \left|B \right| \\&\simeq \left( \frac{\sqrt{t}}{r} \right) ^{\nu } \left|B \right|. \end{aligned}\end{aligned}$$

This, together with the fact that $\left|R \right| \le \left|R_{\sqrt{s + t}} \right|$ gives4.1.3For $R \in {\mathcal {R}}_{j}$, since $d(B,R) \ge (2^{j} - 1) \sqrt{t} \simeq (2^{j} - 1) \sqrt{s + t}$ for $j \ge 1$, we have

4.1.4 $$\begin{aligned} \left( 1 + \frac{d(B,R)^{2}}{s + t} \right) ^{-\frac{\nu + 1}{2}} \lesssim 2^{-j(\nu + 1)}. \end{aligned}$$

Let $\Omega = S_j({\widetilde{B}})$ and ${\mathcal {R}}_{j}$ as defined above in this proof. The inclusion $\Omega \subset 2^{j + 1} {\widetilde{B}} \subset 2^{j + 2}R$ holds for any $R \in {\mathcal {R}}_{j}$ and $j \in {\mathbb {N}}$. Thus Lemma 2.8 implies thatApplying this estimate and (4.1.4–4.1.3) gives us our result. $\square$

Using the previous lemma, the following result can then be proved using an argument identical to the first estimate of [8, Lem. 4.1].

#### Lemma 4.3

Fix $N \in {\mathbb {N}}$ with $N > \mathrm {max}(3 \nu /2 + 1, N_{0})$. For any ball *B* of radius $r(B) > 0$ and $t > r(B)^{2}$ we have4.1.5

Let $S^{\#}$ denote the maximal operatorThis operator was introduced in [8] and formed an important part of their sparse domination argument.

#### Proposition 4.4

For every $$x \in M$$,

$$\begin{aligned} S^{*}f(x) \lesssim S^{\#}f(x) + {\mathcal {M}}_{p_{0}}f(x). \end{aligned}$$

#### Proof

For $x \in M$ and ball $B \subset M$ containing *x*, the triangle inequality impliesFor the first term, apply Minkowski’s inequality followed by Lemma 4.3 to obtainFor the second term,We thus obtain the pointwise estimate (4.4). $\square$

### Cancellation of *S* with respect to *L*

As the operator ${\mathcal {M}}_{p_{0}}$ is $L^{2}$-bounded and weak-type $(p_{0},p_{0})$, the pointwise bound of the previous section implies that in order to prove Theorem 4.1 it will be sufficient to show that $S^{\#}$ is $L^{2}$-bounded and weak-type $(p_{0},p_{0})$. According to [8, Prop. 4.6], $S^{\#}$ will be $L^{2}$-bounded and weak-type $(p_{0},p_{0})$ if *S* satisfies the assumptions of [8]. The only assumption from [8] that is not included in our hypotheses is Assumption (b) of [8], the cancellative property of *S* with respect to *L*. Instead, for us, the cancellation has been imposed upon the constituent operators ${\mathcal {Q}}_{t}$. In this section it will be proved that cancellation on ${\mathcal {Q}}_{t}$ with respect to *L* implies cancellation on *S* with respect to *L*.

#### Proposition 4.5

There exists ${\widetilde{N}}_{0} \ge N_{0}$ such that for all integers $N \ge {\widetilde{N}}_{0}$, $s > 0$ and balls $B_{1}, \, B_{2}$ of radius $\sqrt{s}$,4.2.1for all $f \in L^{p_{0}}(B_{1})$.

#### Proof

For $I \subset [0,\infty )$, define the operator

$$\begin{aligned} S^{I} f(x) := \left( \int _{I} \left|{\mathcal {Q}}_{t} f \right|^{2} \frac{ \, {\text {d}}{t}}{t} \right) ^{1/2}. \end{aligned}$$

In order to prove (4.2.1), it is sufficient to show that a similar estimate holds for the operators $S^{[0,s]}$ and $S^{[s,\infty )}$.

For $I \subset [0,\infty )$, Minkowski’s inequality implies thatFrom Assumption 1.2(c) and the growth property (1.0.4), we haveThe property that $\varphi (a) \le 1$ for $a \le 1$ then gives4.2.2In order to prove the desired off-diagonal estimate, it is then sufficient to prove

4.2.3 $$\begin{aligned} \begin{aligned} A_{I}&:= \int _{I} \frac{t^{2 A_{0}}s^{2N}}{(t + s)^{2 (A_{0} + N)}} \left( 1 + \frac{d(B_{1},B_{2})^{2}}{t + s} \right) ^{-(\nu + 1)} \, \frac{ \, {\text {d}}{t}}{t} \\&\lesssim \left( 1 + \frac{d(B_{1},B_{2})^{2}}{s} \right) ^{- (\nu + 1)}, \end{aligned}\end{aligned}$$

for both intervals $I = [0,s]$ and $I = [s,\infty )$. Consider first the interval $I = [0,s]$. For *t* contained in [0, *s*] we will have $t + s \le 2 s$, and therefore,

$$\begin{aligned} \left( 1 + \frac{d(B_{1},B_{2})^{2}}{t + s} \right) ^{- (\nu + 1)} \lesssim \left( 1 + \frac{d(B_{1},B_{2})^{2}}{s} \right) ^{- (\nu + 1)}. \end{aligned}$$

This gives

$$\begin{aligned}\begin{aligned} A_{I}&\lesssim \left( 1 + \frac{d(B_{1},B_{2})^{2}}{s} \right) ^{- (\nu + 1)} \int _{0}^{s} \frac{t^{2 A_{0}} s^{2N}}{(t + s)^{2(A_{0} + N)}} \, \frac{ \, {\text {d}}{t}}{t} \\&\le \left( 1 + \frac{d(B_{1},B_{2})^{2}}{s} \right) ^{-(\nu + 1)} \frac{1}{s} \int ^{s}_{0} \, {\text {d}}{t} \\&= \left( 1 + \frac{d(B_{1},B_{2})^{2}}{s} \right) ^{-(\nu + 1)}. \end{aligned}\end{aligned}$$

Applying this to (4.2.2) produces the desired off-diagonal bounds for the operator $S^{[0,s]}$.

Next, let’s prove off-diagonal bounds for the operator $S^{[s,\infty )}$. Suppose first that $s > d(B_{1},B_{2})^{2}$. When this occurs, note that

4.2.4 $$\begin{aligned} \left( 1 + \frac{d(B_{1},B_{2})^{2}}{s} \right) ^{-(\nu + 1)} \simeq 1. \end{aligned}$$

We then have,

$$\begin{aligned}\begin{aligned} A_{I}&\le \int ^{\infty }_{s} \frac{t^{2 A_{0}} s^{2 N}}{(t + s)^{2(A_{0} + N)}} \, \frac{ \, {\text {d}}{t}}{t} \\&\le s^{2 N} \int ^{\infty }_{s} \frac{1}{(t + s)^{2 N + 1}} \, {\text {d}}{t} \\&\simeq 1 \\&\simeq \left( 1 + \frac{d(B_{1},B_{2})^{2}}{s} \right) ^{- (\nu + 1)}. \end{aligned}\end{aligned}$$

Applying this to (4.2.2) produces the desired off-diagonal estimates for $S^{[s,\infty )}$.

Finally, we must prove off-diagonal decay for $S^{[s,\infty )}$ for the case $s \le d(B_{1},B_{2})^{2}$. We have,

$$\begin{aligned}\begin{aligned} A_{I}&= \int ^{\infty }_{s} \frac{t^{2 A_{0}} s^{2 N}}{(t + s)^{2 (A_{0} + N)}} \left( 1 + \frac{d(B_{1},B_{2})^{2}}{t + s} \right) ^{-(\nu + 1)} \, \frac{ \, {\text {d}}{t}}{t} \\&\le \frac{s^{2 N}}{d(B_{1},B_{2})^{2 (\nu + 1)}} \int ^{\infty }_{s} \frac{ \, {\text {d}}{t}}{(t + s)^{2 N + 1 - (\nu + 1)}}. \end{aligned}\end{aligned}$$

Select ${\widetilde{N}}_{0} \ge N_{0}$ large enough so that $N \ge {\widetilde{N}}_{0}$ implies $2N > \nu + 1$. Then,

$$\begin{aligned}\begin{aligned} A_{I}&\lesssim \frac{s^{\nu + 1}}{d(B_{1},B_{2})^{2 (\nu + 1)}} \\&\lesssim \left( 1 + \frac{d(B_{1},B_{2})^{2}}{s} \right) ^{-(\nu + 1)}, \end{aligned}\end{aligned}$$

where the last line follows from the condition $s \le d(B_{1},B_{2})^{2}$. Applying this to (4.2.2) completes our proof. $\square$

The below corollary, in combination with the pointwise estimate Proposition 4.4, completes the proof of Theorem 4.1.

#### Corollary 4.6

[8, Prop. 4.6] The maximal function $S^{\#}$ is bounded on $L^{2}$, and weak-type $(p_{0},p_{0})$.

## Sparse Bounds

In this section we prove Theorem 1.7. Since *f* has compact support, without loss of generality we can assume that its support is contained in a bounded set $E\subset M$. By the Lemma 2.3, there exists $\alpha \ge 1$ and a partition $\mathscr {P}$ of *M* of dyadic cubes such that $\alpha Q \supseteq \mathrm {supp}f$ for every $Q \in \mathscr {P}$. Then

$$\begin{aligned} \int _{M} |Sf|^2 g \, {\text {d}}{\mu } = \sum _{Q \in \mathscr {P}} \int _{Q} |S f|^2 g \, {\text {d}}{\mu } = \sum _{Q \in \mathscr {P}} \int _{Q} |S (f \mathbb {1}_{\alpha Q})|^2 g \, {\text {d}}{\mu } . \end{aligned}$$

We are not concerned with the particular value of $\alpha$, so we will fix $\alpha = 5$ in the following and assume that this value works for the covering lemma. Then, it is enough to show the existence of a sparse collection ${\mathcal {S}}_0$ inside a fixed cube $Q_0$ such thatWe will decompose our quantity in different terms: all will be controlled by the averages of *f* and *g* but one. This last term is where *f* assumes a large value and it is similar to the original quantity but on a smaller scale. We can then iterate the decomposition, which terminates since the measure of the set we are decomposing shrinks geometrically at each iteration.

### Decomposition

Denote by $\ell (P)$ the side length of the dyadic cube *P*. Let us consider the (localised) dyadic version of the operator introduced in Sect. 4,For a positive $\eta$ to be fixed later, consider the setSince the operators ${\mathcal {M}}^*_{Q_0,p_0}$ and $S_{Q_0}^{*}$ are weak-type $(p_0,p_0)$, as shown in Sect. 4, there exists $\eta > 0$ such that $|E(Q_0)|\le \frac{1}{2} |Q_0|$. Decompose our form as

$$\begin{aligned} \int _{Q_0} (Sf)^2 g \, {\text {d}}{\mu } = \int _{Q_0 \setminus E(Q_0)} (Sf)^2 g \, {\text {d}}{\mu } + \int _{E(Q_0)} (Sf)^2 g \, {\text {d}}{\mu } =:\text {I} + \text {II} \end{aligned}$$

Term I is controlled by using Lebesgue differentiation theorem as in [8, Lem. 4.4] since $|Sf(x) |^2 \le |S^{*}_{Q_0}f(x) |^2$ for $\mu$-almost every *x*. Thus, for $x \in Q_0\setminus E(Q_0)$ we haveConsider term II. Let $\mathscr {E} :=\{P\}_{P\in \mathscr {D}}$ be a covering of $E(Q_0)$ with maximal dyadic cubes. Then

$$\begin{aligned} \int _{E(Q_0)} (Sf)^2 g \, {\text {d}}{\mu }&= \sum _{P\in \mathscr {E}} \int _{P} (Sf)^2 g \, {\text {d}}{\mu } \\&= \sum _{P\in \mathscr {E}} \int _{P} \int _0^{\ell (P)^2} |{\mathcal {Q}}_t f(x)|^2 \frac{ \, {\text {d}}{t}}{t} g \, {\text {d}}{\mu } \\&\quad + \sum _{P\in \mathscr {E}} \int _{P} \int _{\ell (P)^2}^\infty |{\mathcal {Q}}_t f(x)|^2 \frac{ \, {\text {d}}{t}}{t} g \, {\text {d}}{\mu } \\&=:\text {II}_{<} + \text {II}_{>} . \end{aligned}$$

For each *P* in the covering, we write $f = f_{\mathsf {in}} + f_{\mathsf {out}}$, where $f_{\mathsf {in}} :=f \mathbb {1}_{5P}$ and $f_{\mathsf {out}} :=f \mathbb {1}_{(5P)^\complement }$. Then each term in $\text {II}_{<}$ is itself decomposed into three terms 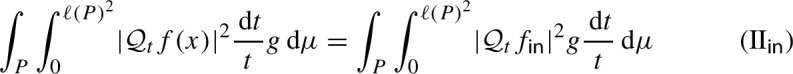

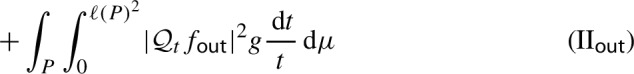

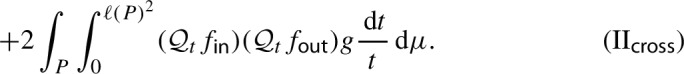


Term ($\text {II}_{\mathsf {in}}$) goes into the iteration. Terms ($\text {II}_{\mathsf {out}}$) and ($\text {II}_{\mathsf {cross}}$) are controlled by using Fubini and applying off-diagonal estimates as in the following lemma.

#### Lemma 5.1

For a given dyadic cube *P*, let $S_k(P) :=2^{k+1}P\setminus 2^{k}P$ for $k\ge 2$. Then for any $t >0$,5.1.15.1.2

#### Proof of Lemma 5.1

The proof follows the one in [8, Thm. 5.7]. For $f_{\mathsf {in}} = f \mathbb {1}_{5P}$, let ${\mathcal {R}}_0$ be a collection of finite overlapping balls *R* of radius $\sqrt{t}$ covering 5*P*. By linearity of the operators, the triangle inequality, off-diagonal estimates for ${\mathcal {Q}}_t$ with $\rho (x)= (1 + |x|^2)^{-(\nu + 1)}$ and Remark 2.9 we haveSince $5 P \subseteq \frac{15 \ell (P)}{\sqrt{t}}R$, Lemma 2.8 implieswhich proves (5.1.1).

For $f_{\mathsf {out}} = f \mathbb {1}_{(5P)^\complement }$, decompose *f* on the squared annuli $S_k = S_k(P)$. Let ${\mathcal {R}}_k$ be the covering of $S_k$ with finite overlapping balls *R* of radius $\sqrt{t}$. Linearity of the operators ${\mathcal {Q}}_{t}$, the triangle inequality and off-diagonal estimates for ${\mathcal {Q}}_t$ imply thatwhere we used that the function $\rho$ is monotone decreasing and $d(P,R) \ge d(P,S_k)$. The last inequality follows by applying Lemma 2.8, since $S_k(P) \subseteq 2^{k} P \subseteq \frac{2^{k+1} \ell (P)}{\sqrt{t}}R$.

Finally, we have enough decay from the remaining product, since

$$\begin{aligned} \rho \Big (\frac{d(P,S_k)}{\sqrt{t}}\Big ) \left( \frac{2^{k+1}\ell (P)}{\sqrt{t}}\right) ^\nu \lesssim \Big ( \frac{2^k \ell (P)}{\sqrt{t}}\Big )^{-\nu -2} \end{aligned}$$

This follows because $d(P,S_k) = d(P,2^{k+1}P\setminus 2^k P)$ is comparable with $2^k \ell (P)$ and the function $\rho (x)= (1 + |x|^2)^{-(\nu + 1)}$ decays faster than $x^\nu$ for $x \gg 1$. This proves estimate (5.1.2). $\square$

We will use Lemma 5.1 to control the different terms left in the decomposition.

#### Remark 5.2

The geometric sum in (5.1.2) is controlled using the stopping condition: the integral over $S_k$ is bounded by the integral over the ball $2^{k+1}P$, sowhere we used that *P* is a maximal cube covering *E*. Similarly for the average on 5*P*:

#### Remark 5.3

(Control on the $q_0^*$-average of *g*) The sum of the $q_0^*$-averages of *g* is controlled by using Hölder’s inequality in $\ell ^{\frac{q_0}{2}}$. Since $\frac{2}{q_0} = 1- \frac{1}{q_0^*}$, summing over all cubes *P* in $\mathscr {E}$ we obtain5.1.3

### Out Term

Consider ($\text {II}_{\mathsf {out}}$). Applying Fubini and Hölder’s inequality, we haveThe average of *g* is controlled as in (5.1.3). Apply Lemma 5.1 to the first factor:which is controlled as in Remark 5.2. This case is concluded.

### Cross Term

Consider ($\text {II}_{\mathsf {cross}}$). We exchange the integrals, then an application of Hölder’s and Cauchy–Schwarz inequality giveThe off-diagonal estimates for ${\mathcal {Q}}_t$ in Lemma 5.1 applied to $f_{\mathsf {in}}$ and $f_{\mathsf {out}}$ imply thatwhere the last estimate follows as in Remark 5.2.

### Large Scales

Consider $\text {II}_{>}$. Let $P^a$ be the dyadic parent of *P*, so that $\ell (P^a) = 2 \ell (P)$. Then

5.4.1 $$\begin{aligned}&\int _{P} \int _{\ell (P)^2}^\infty |{\mathcal {Q}}_t f(x)|^2 \frac{ \, {\text {d}}{t}}{t} g \, {\text {d}}{\mu } \nonumber \\&\quad = \int _{P} \int _{\ell (P)^2}^{\ell (P^a)^2} |{\mathcal {Q}}_t f(x)|^2 \frac{ \, {\text {d}}{t}}{t} g \, {\text {d}}{\mu } + \int _{P} \int _{\ell (P^a)^2}^\infty |{\mathcal {Q}}_t f(x)|^2 \frac{ \, {\text {d}}{t}}{t} g \, {\text {d}}{\mu } . \end{aligned}$$

In the first term, we exchange the integrals and apply Hölder’s inequalityApplying Lemma 5.1 and using that $\sqrt{t}$ is comparable with $\ell (P)$, we obtainwhich again is controlled as in Remark 5.2. The average of *g* is estimated as in (5.1.3).

The second term in (5.4.1), after applying Hölder’s inequality, is controlled by the maximal truncationWe have shown thatLet ${\mathcal {S}} = \{Q_0\}$. We add all *P* in the sum to ${\mathcal {S}}$ and we repeat the argument on each term in the sum. This iteration gives the desired bound: a sum of averages of *f* and *g* on cubes in the collection ${\mathcal {S}}$. We can choose $\eta >0$ such that $|E(Q)|\le \frac{1}{2} |Q|$ for each $Q\in {\mathcal {S}}$. Then ${\mathcal {S}}$ is sparse since each $Q\in {\mathcal {S}}$ has a subset $F_Q :=Q \setminus E(Q)$ with the property that $\{ F_Q \}_{Q\in {\mathcal {S}}}$ is a disjoint family and $|F_Q|> \frac{1}{2} |Q|$ by construction. $\square$

## Weighted Boundedness

In this section, we provide the proof of Theorem 1.8. We begin by recalling the notation $p^{*} := (p/2)' = \frac{p}{p - 2}$ for $p > 2$. We will also make use of the notation

$$\begin{aligned} \phi (p) := \left( \frac{q_{0}}{p} \right) ' \left( \frac{p}{p_{0}} - 1 \right) + 1 \end{aligned}$$

for $1 \le p_{0}< 2 < q_{0} \le \infty$ and $p \in (p_{0},q_{0})$, which was previously introduced in Sect. 2.3.

### Remark 6.1

Define the critical index ${\mathfrak {p}}$ through

6.0.2 $$\begin{aligned} {\mathfrak {p}} := 2 + p_{0} - \frac{2 p_{0}}{q_{0}} = 2 + \frac{p_{0}}{q_{0}^{*}}. \end{aligned}$$

The critical exponent is the unique $p \in (1,\infty )$ that satisfies the relation $p^{*} = \phi (p)$. It is easy to check that ${\mathfrak {p}}$ is contained in the interval $(2,q_{0})$ and that it satisfies the relation

6.0.3 $$\begin{aligned} \frac{1}{{\mathfrak {p}} - p_{0}} = \left( \frac{q_{0}}{{\mathfrak {p}}} \right) ' \frac{1}{2 q_{0}^{*}}. \end{aligned}$$

Thus, we also have that

$$\begin{aligned} \gamma (p) = \max \left( \frac{1}{p - p_{0}},\left( \frac{q_{0}}{p} \right) ' \frac{1}{2 q_{0}^{*}} \right) = \left( \frac{q_{0}}{p} \right) ' \frac{1}{2 q_{0}^{*}} \end{aligned}$$

if and only if $p \ge {\mathfrak {p}}$, and $\gamma (p) = (p - p_{0})^{-1}$ if and only if $p \le {\mathfrak {p}}$. In [8], the critical exponent for the linear sparse domination is $1 + p_0/q_0'$ which is evidently analogous to the definition of ${\mathfrak {p}}$ in (6.0.2).

### Proof of Theorem 1.8

Fix $p \in (2,q_{0})$. Notice that by (2.3.1),

$$\begin{aligned} \left[ w^{(\frac{q_{0}}{p})'} \right] _{A_{\phi (p)}} \le \left( \left[ w \right] _{A_{\frac{p}{p_{0}}}} \cdot \left[ w \right] _{RH_{(\frac{q_{0}}{p})'}} \right) ^{(\frac{q_{0}}{p})'}. \end{aligned}$$

This tells us that in order to prove the estimate in Theorem 1.8, it is sufficient to demonstrate the stronger estimate6.1.1By Theorem 2.2, for each $P \in {\mathcal {S}}$ there will exist ${\bar{P}} \in \mathscr {D}$ for which $5 P \subset {\bar{P}}$ and $\left|{\bar{P}} \right| \lesssim \left|5 P \right|$. Then $\left|{\bar{P}} \right| \lesssim \left|P \right|$ by the doubling property of dyadic cubes. As the collection ${\mathcal {S}}$ is sparse, there must exist a collection of disjoint sets $\left\{ E_{P} \right\} _{P \in {\mathcal {S}}}$ such that $E_{P} \subset P$ and $\left|P \right| \lesssim \left|E_{P} \right|$ for all $P \in {\mathcal {S}}$. We, therefore, have

$$\begin{aligned} \left|{\bar{P}} \right| \lesssim \left|E_{P} \right|. \end{aligned}$$

Define the weight $v := w^{(q_{0}/p)'}$ and $r := \phi (p) = \left( \frac{q_{0}}{p} \right) ' \left( \frac{p}{p_{0}} - 1 \right) + 1$. Set *u* to be the dual weight of *v* in $A_{r}$, $u := v^{1 - r'}$. We haveandApplying these two relations to our sparse form leads to6.1.2*Case 1: *$p \ge {\mathfrak {p}}$. Note that by Remark 6.1 this assumption is equivalent to assuming that $\gamma (p) = \left( \frac{q_{0}}{p} \right) ' \frac{1}{2 q_{0}^{*}}$. If we define

$$\begin{aligned} \kappa (p) := \frac{2}{p_{0}} - \frac{r - 1}{q_{0}^{*}}, \end{aligned}$$

then our assumption is also equivalent to the condition $\kappa (p) \le 0$. The fact that *u* is the conjugate weight of *v* in $A_{r}$ implies that for $P \in {\mathcal {S}}$,This estimate can be applied to (6.1.2) to produce6.1.3Since $\left|{\bar{P}} \right| \lesssim \left|E_{P} \right|$ and $\kappa (p) \le 0$,For $\lambda := (1 - \kappa (p))^{-1}$ notice that

$$\begin{aligned} \frac{\lambda }{p/2} + \frac{\lambda }{p^{*}} - \lambda \kappa (p) = \lambda (1 - \kappa (p)) = 1. \end{aligned}$$

Also, it is straightforward to check by substituting in the definition $u :=v^{1 - r'}$ that the constant function 1 can be decomposed as

$$\begin{aligned} 1 = u^{\frac{1}{p/2}} v^{\frac{1}{p^{*}}} u^{- \kappa (p)}. \end{aligned}$$

From this, Hölder’s inequality implies

$$\begin{aligned}\begin{aligned} \left|E_{P} \right|&= \int _{E_{P}} u^{\frac{\lambda }{p/2}} v^{\frac{\lambda }{p^{*}}} u^{- \lambda \kappa (p)} \, {\text {d}}{\mu } \\&\le \left( \int _{E_{P}} u \right) ^{\frac{\lambda }{p/2}} \left( \int _{E_{P}} v \right) ^{\frac{\lambda }{p^{*}}} \left( \int _{E_{P}} u \right) ^{-\lambda \kappa (p)}, \end{aligned}\end{aligned}$$

and, therefore, raising to the power $1/\lambda$ produces

$$\begin{aligned} u(E_P)^{\kappa (p)} \left|E_P \right|^{1/\lambda } \le u(E_P)^{2/p} v(E_P)^{1/p^*}. \end{aligned}$$

Applying this estimate to (6.1.3) and Hölder’s inequality leads toSince $p > p_{0}$ the operator ${\mathcal {M}}^{\mathscr {D}}_{p_0,u}$ is bounded on $L^{p}(u \, {\text {d}}{\mu })$ with constant independent of *u*. Similarly, since $p^{*} > q_{0}^{*}$ the operator ${\mathcal {M}}^{\mathscr {D}}_{q_{0}^{*},v}$ is bounded on $L^{p^{*}}(v \, {\text {d}}{\mu })$ with constant independent of *v*. These observations lead to the estimateFrom this estimate and the relation $\frac{1}{q_{0}^{*}} = \frac{2 \gamma (p)}{(q_{0}/p)'}$ it is clear that (6.1.1) will follow if it can be shown that $u^{1 - \frac{p}{p_{0}}} = w$ and $v^{1 - \frac{p^{*}}{q_{0}^{*}}} = \sigma$. Let’s first prove that $u^{1 - \frac{p}{p_{0}}} = w$. As *u* is defined through $u = v^{1 - r'} = w^{\left( \frac{q_{0}}{p} \right) '(1 - r')}$ we have

$$\begin{aligned} u^{1 - \frac{p}{p_{0}}} = w^{\left( \frac{q_0}{p} \right) '(1 - r') \left( 1 - \frac{p}{p_{0}} \right) } = w^{(1 - r')(1-r)} = w, \end{aligned}$$

where the second equality follows from the definition $r = \phi (p)$.

It remains to show that $v^{1 - \frac{p^{*}}{q_{0}^{*}}} = \sigma$. The definitions $v = w^{\left( \frac{q_{0}}{p} \right) '}$ and $\sigma = w^{1 - p^{*}}$ transform this relation into

$$\begin{aligned} w^{\left( \frac{q_0}{p} \right) ' \left( 1 - \frac{p^{*}}{q_{0}^{*}} \right) } = w^{1 - p^{*}}. \end{aligned}$$

It must, therefore, be proved that

$$\begin{aligned} \left( \frac{q_0}{p} \right) ' \left( 1 - \frac{p^{*}}{q_{0}^{*}} \right) = 1 - p^{*}. \end{aligned}$$

This is equivalent to showing that

$$\begin{aligned} \left( \frac{q_{0}}{q_{0} - p} \right) \left( 1 - \frac{p(q_{0} - 2)}{q_{0}(p - 2)} \right) = 1 - \frac{p}{p - 2}. \end{aligned}$$

Through simple algebraic manipulation, it is easy to check that the two sides of the above equality indeed coincide. This validates the relation $v^{1 - \frac{p^{*}}{q_{0}^{*}}} = \sigma$ and completes our proof for $p \ge {\mathfrak {p}}$.

*Case 2: *$p \le {\mathfrak {p}}$. This assumption is equivalent to assuming that $\gamma (p) = \frac{1}{p - p_{0}}$ or, alternatively, $\kappa (p) \ge 0$. Define

$$\begin{aligned} {\bar{\kappa }}(p) := \frac{1}{q_{0}^{*}} - \frac{2}{p_{0}(r - 1)}. \end{aligned}$$

ThenCombining this with (6.1.2) gives6.1.4It is clear that ${\bar{\kappa }}(p) = - (r - 1)^{-1}\kappa (p) \le 0$. It then follows from this and $\left|{\bar{P}} \right| \lesssim \left|E_{P} \right|$ thatDefine ${\bar{\lambda }} := (1 - {\bar{\kappa }}(p))^{-1}$. Then we have

$$\begin{aligned} \frac{{\bar{\lambda }}}{p/2} + \frac{{\bar{\lambda }}}{p^{*}} - {\bar{\lambda }} {\bar{\kappa }}(p) = {\bar{\lambda }}(1 - {\bar{\kappa }}(p)) = 1. \end{aligned}$$

Also, it is straightforward to check by substituting in the definition $u = v^{1 - r'}$ that the constant function 1 can be decomposed as

$$\begin{aligned} 1 = u^{\frac{1}{p/2}} v^{\frac{1}{p^{*}}} v^{- {\bar{\kappa }}(p)}. \end{aligned}$$

Hölder’s inequality then implies

$$\begin{aligned}\begin{aligned} \left|E_{P} \right|&= \int _{E_{P}} u^{\frac{{\bar{\lambda }}}{p/2}} v^{\frac{{\bar{\lambda }}}{p^{*}}} v^{- {\bar{\lambda }} {\bar{\kappa }}(p)} \, {\text {d}}{\mu } \\&\le \left( \int _{E_{P}} u \right) ^{\frac{{\bar{\lambda }}}{p/2}} \left( \int _{E_{P}} v \right) ^{\frac{{\bar{\lambda }}}{p^{*}}} \left( \int _{E_{P}} v \right) ^{- {\bar{\lambda }} {\bar{\kappa }}(p)}. \end{aligned}\end{aligned}$$

Raising to the power $1/{\bar{\lambda }}$ produces

$$\begin{aligned} v(E_P)^{{\bar{\kappa }}(p)} \left|E_P \right|^{1/{\bar{\lambda }}} \le u(E_P)^{2/p} v(E_P)^{1/p^*}. \end{aligned}$$

Applying this to (6.1.4), since $1/{\bar{\lambda }} = 1 - {\bar{\kappa }}(p)$, yieldsAfter noting that $\frac{2}{p_{0}(r - 1)} = \frac{2 \gamma (p)}{(q_{0}/p)'}$ the proof of (6.1.1) then proceeds in an identical manner to the case $p \ge {\mathfrak {p}}$. $\square$

### Proof of Corollary 1.9

We start by noting that

$$\begin{aligned}\begin{aligned} \left\Vert S f \right\Vert _{L^{{\mathfrak {p}}}(w)}^{2}&= \left\Vert (S f)^{2} \right\Vert _{L^{\frac{{\mathfrak {p}}}{2}}(w)} \\&= \sup _{\left\Vert g \right\Vert _{L^{{\mathfrak {p}}^{*}}(\sigma )} = 1} \left|\langle (Sf)^{2}, g \rangle \right|, \end{aligned}\end{aligned}$$

where $\sigma := w^{1 - \left( \frac{{\mathfrak {p}}}{2} \right) '} = w^{1 - {\mathfrak {p}}^{*}}$ is the $A_{\frac{{\mathfrak {p}}}{2}}$-conjugate weight of *w*. Thus, in order to prove the desired result, it is sufficient to demonstrate the estimate

6.2.1 $$\begin{aligned} \left|\langle (S f)^{2}, g \rangle \right| \lesssim \left[ w^{(q_{0}/{\mathfrak {p}})'} \right] _{A_{\phi ({\mathfrak {p}})}}^{\frac{1}{q_{0}^{*}}} \left\Vert f \right\Vert _{L^{{\mathfrak {p}}}(w)}^{2} \left\Vert g \right\Vert _{L^{{\mathfrak {p}}}(\sigma )}. \end{aligned}$$

For the critical index ${\mathfrak {p}}$ this is an easy consequence of Theorem 1.7, estimate (6.1.1) and a density argument.

Applying the sharp restricted range extrapolation (Theorem 2.15) yields that for any $p \in (p_{0},q_{0})$ and weight $w \in A_{\frac{p}{p_{0}}} \cap RH_{(\frac{q_{0}}{p})'}$,

6.2.2 $$\begin{aligned} \left\Vert S f \right\Vert _{L^{p}(w)} \lesssim \left[ w^{(\frac{q_{0}}{p})'} \right] ^{\beta (p,{\mathfrak {p}})/(2 q_{0}^{*})}_{A_{\phi (p)}} \end{aligned}$$

where $$\beta (p,{\mathfrak {p}}) = \max \left( 1, \frac{(q_{0} - p)({\mathfrak {p}} - p_{0})}{(q_{0} - {\mathfrak {p}})(p - p_{0})} \right) $$.

We check that this matches the power $\gamma (p)$ in Corollary 1.9. Let $\omega (p) :=(q_{0} - p)/(p - p_{0})$ for $p\in (p_0,q_0)$. Then $\beta (p,{\mathfrak {p}}) = \max ( 1, \omega (p) /\omega ({\mathfrak {p}}) )$. Since $\omega (p)$ is decreasing in *p* and $\beta ({\mathfrak {p}},{\mathfrak {p}}) = 1$, then $\beta (p,{\mathfrak {p}}) = 1$ for $p \in [{\mathfrak {p}},q_0)$. In this range of exponent we have

$$\begin{aligned} \left\Vert S f \right\Vert _{L^{p}(w)} \lesssim \left[ w^{(\frac{q_{0}}{p})'} \right] ^{1/(2 q_{0}^{*})}_{A_{\phi (p)}} \lesssim \left( \left[ w \right] _{A_{\frac{p}{p_{0}}}} \cdot \left[ w \right] _{RH_{(\frac{q_{0}}{p})'}} \right) ^{(\frac{q_{0}}{p})'/(2 q_{0}^{*})}, \end{aligned}$$

where the last inequality is the bound on the weight characteristic given in (2.3.1).

When $p < {\mathfrak {p}}$, instead $\beta (p,{\mathfrak {p}}) = \omega (p)/\omega ({\mathfrak {p}})$. Using the identity (6.0.3) for ${\mathfrak {p}}$, one can see that $\omega ({\mathfrak {p}}) \cdot 2q_0^* = q_0$. This immediately gives

$$\begin{aligned} \frac{\beta (p,{\mathfrak {p}})}{2q_0^*} = \frac{\omega (p)}{\omega ({\mathfrak {p}}) \cdot 2q_0^*} = \frac{\omega (p)}{q_0} = \frac{1}{\left( q_0/p \right) '} \frac{1}{p-p_0}. \end{aligned}$$

Then (6.2.2) followed by (2.3.1) implies that for $p \in (p_0,{\mathfrak {p}})$

$$\begin{aligned} \left\Vert S f \right\Vert _{L^{p}(w)} \lesssim \left[ w^{(\frac{q_{0}}{p})'} \right] ^{\frac{1}{\left( q_0/p \right) '} \frac{1}{p-p_0}}_{A_{\phi (p)}} \lesssim \left( \left[ w \right] _{A_{\frac{p}{p_{0}}}} \cdot \left[ w \right] _{RH_{(\frac{q_{0}}{p})'}} \right) ^{1/(p-p_0)} \end{aligned}$$

The exponent in the above inequality matches the hypothesised exponent of (2.3.1), allowing us to conclude our proof. $\square$

## Sharpness of the Sparse form for $p>2$

In this section we will use the notation $\sim$ to indicate asymptotic behaviour and we will work in ${\mathbb {R}}$ with the Lebesgue measure. The sharpness in Theorem 1.8 is a consequence of the following proposition. The proof, although different, follows the reasoning in [8, §7].

### Proposition 7.1

For $p\in (2,q_0)$, there exists a sparse collection ${\mathcal {S}}$ and for every $0<\epsilon <1$, there exist sequences of functions $f_\epsilon$ and $g_\epsilon$ and weights $w_\epsilon$ such that7.0.3as $\epsilon \rightarrow 0$, where

$$\begin{aligned} \gamma (p) :=\max \left( \frac{1}{p - p_{0}}, \left( \frac{q_{0}}{p} \right) ' \frac{1}{2q_{0}^{*}} \right) \,\,\, \text { and } \,\,\, \sigma _\epsilon :=w_{\epsilon }^{1-(p/2)'}. \end{aligned}$$

### Proof

The proof is divided into two cases, the case where $p\le {\mathfrak {p}}$ and the case where $p\ge {\mathfrak {p}}$. In both of them, the sparse collection considered is ${\mathcal {S}}=\{I_{n}:=[0,2^{-n}]\, :\, \text {for } n\in {\mathbb {N}}\}$.

For $2<p\le {\mathfrak {p}}$ and fixed $0<\epsilon <1$, consider the functions

$$\begin{aligned} f_\epsilon (x)&:=x^{-\frac{1}{p_0}+\epsilon }\chi _{[0,1]}\\ g_\epsilon (x)&:=x^{-\frac{1}{p_0^{*}}+\epsilon }\chi _{[0,1]}\\ w_\epsilon (x)&:=|x|^{\frac{p}{p_0}-1-\epsilon }\chi _{[0,1]}\\ \sigma _{\epsilon }(x)&= |x|^{\left( \frac{p}{p_0}-1-\epsilon \right) (1-p^*)}\chi _{[0,1]}, \end{aligned}$$

where $\sigma _{\epsilon }$ is the dual weight to $w_\epsilon$ in $A_{p/2}$.

Thenas $\epsilon \rightarrow 0$. The left hand side of (7.0.3) follows the asymptotic behaviourFor power weights, the asymptotics of the $A_p$ and $RH_q$ characteristics are well understood, see for instance [12]. Therefore, as $\epsilon \rightarrow 0$, we have

$$\begin{aligned}&\left[ w_\epsilon \right] _{A_{\frac{p}{p_{0}}}} \sim \epsilon ^{-\left( \frac{p}{p_0}-1\right) },\\&\left[ w_\epsilon \right] _{RH_{(\frac{q_{0}}{p})'}} \sim 1. \end{aligned}$$

Moreover we compute the norms on the right hand side of (7.0.3). We have

$$\begin{aligned} \left\Vert f_\epsilon \right\Vert _{L^{p}(w_\epsilon )}&= \left( \int _{0}^{1} x^{\frac{-p}{p_0}+p\epsilon } x^{\frac{p}{p_0}-1-\epsilon } \, {\text {d}}{x}\right) ^{1/p}\\&=\left( \int _{0}^{1} x^{-1+(p-1)\epsilon } \, {\text {d}}{x}\right) ^{1/p}\sim \epsilon ^{-1/p},\\ \end{aligned}$$

as $$\epsilon \rightarrow 0$$.

$$\begin{aligned} \left\Vert g_{\epsilon } \right\Vert _{L^{(p/2)'}(\sigma _\epsilon )}&= \left( \int _{0}^{1} x^{\frac{-p^{*}}{p_0^{*}}+p^{*}\epsilon } x^{\left( \frac{p}{p_0}-1-\epsilon \right) \left( 1-p^{*}\right) } \, {\text {d}}{x}\right) ^{1/p^{*}} \\&= \left( \int _{0}^{1} x^{-1+(2p^{*}-1)\epsilon -\frac{p^{*}}{p_0^{*}}+\frac{p}{p_0}-\frac{pp^{*}}{p_0}+p^{*}} \, {\text {d}}{x}\right) ^{1/p^{*}}. \end{aligned}$$

Using the definition of $p^{*}$ and $p_{0}^{*}$, we note $-\frac{p^{*}}{p_0^{*}}+\frac{p}{p_0}-\frac{pp^{*}}{p_0}+p^{*}=0$, therefore,

$$\begin{aligned} \left\Vert g_{\epsilon } \right\Vert _{L^{(p/2)'}(\sigma _\epsilon )}= \left( \int _{0}^{1} x^{-1+(2p^{*}-1)\epsilon } \, {\text {d}}{x}\right) ^{1/p^{*}}\sim \epsilon ^{-1/p^{*}}, \end{aligned}$$

as $$\epsilon \rightarrow 0$$.

We conclude that the right hand side of (7.0.3) behaves as $\epsilon ^{-\left( \frac{p}{p_0}-1\right) \left( \frac{2}{p-p_0}\right) }\epsilon ^{-2/p} \epsilon ^{-1/p^{*}}=\epsilon ^{-1}\epsilon ^{-2/p_0}$ as $\epsilon \rightarrow 0$, which is exactly the asymptotic behaviour of the left hand side of (7.0.3) as desired.

For ${\mathfrak {p}}\le p<q_0$ and fixed $0<\epsilon <1$, consider the functions

$$\begin{aligned} f_\epsilon (x)&:=x^{-\frac{1}{q_0}+\epsilon }\chi _{[0,1]}\\ g_\epsilon (x)&:=x^{-\frac{1}{q_0^{*}}+\epsilon }\chi _{[0,1]}\\ \sigma _\epsilon (x)&:=|x|^{\frac{p^*}{q_0^*}-1-\epsilon }\chi _{[0,1]}\\ w_\epsilon&= |x|^{\left( \frac{p^*}{q_0^*}-1-\epsilon \right) \left( 1-p/2\right) }, \end{aligned}$$

where $\sigma _{\epsilon }$ is the dual weight to $w_\epsilon$ in $A_{p/2}$.

Thenas $\epsilon \rightarrow 0$. And the right hand side of (7.0.3) follows the asymptotic behaviourFor the power weights $w_\epsilon$, as $\epsilon \rightarrow 0$, we have

$$\begin{aligned}&\left[ w_\epsilon \right] _{A_{\frac{p}{p_{0}}}} \sim 1,\\&\left[ w_\epsilon \right] _{RH_{(\frac{q_{0}}{p})'}} \sim \epsilon ^{\frac{-1}{(q_0/p)'}}. \end{aligned}$$

Moreover we compute

$$\begin{aligned} \left\Vert g_{\epsilon } \right\Vert _{L^{(p/2)'}(\sigma _\epsilon )}=\left( \int _{0}^{1} x^{-1+(p^*-1)\epsilon } \, {\text {d}}{x}\right) ^{1/p^{*}}\sim \epsilon ^{-1/p^{*}}, \end{aligned}$$

as $$\epsilon \rightarrow 0$$, and

$$\begin{aligned} \left\Vert f_\epsilon \right\Vert _{L^{p}(w_\epsilon )}&= \left( \int _{0}^{1}x^{\frac{-p}{q_0}+\epsilon p}x^{\left( \frac{p^{*}}{q_0^{*}}-1-\epsilon \right) \left( 1-\frac{p}{2} \right) } \, {\text {d}}{x}\right) ^{1/p}\\&= \left( \int _{0}^{1}x^{\left( \frac{3p}{2}-1\right) \epsilon -1 -\frac{p}{q_0} +\frac{p^{*}}{q_0^{*}}-\frac{pp^{*}}{2q_0^{*}} +\frac{p}{2} } \, {\text {d}}{x}\right) ^{1/p}.\\ \end{aligned}$$

Using the definition of $p^{*}$ and $q_{0}^{*}$, we note $-\frac{p}{q_0}+\frac{p^{*}}{q_0^{*}}-\frac{pp^{*}}{2q_0^{*}}+\frac{p}{2}=0$, therefore,

$$\begin{aligned} \left\Vert f_\epsilon \right\Vert _{L^{p}(w_\epsilon )} =\left( \int _{0}^{1} x^{(3p/2-1)\epsilon -1} \, {\text {d}}{x}\right) ^{1/p}\sim \epsilon ^{-1/p}. \end{aligned}$$

We conclude the right hand side of (7.0.3) behaves as $\epsilon ^{-1/q_0^*}\epsilon ^{-2/p} \epsilon ^{-1/p^{*}}=\epsilon ^{-1}\epsilon ^{-1/q_0^*}$ as $\epsilon \rightarrow 0$, which is exactly the asymptotic for the left hand side of (7.0.3) as desired. $\square$

### Upper Bound on Asymptotic Behaviour

In this section we discuss the connection between sharp weighted estimates for an operator *T* and the asymptotic behaviour of its unweighted norm $\Vert T \Vert _{L^p\rightarrow L^p}$. We recall the definition of $\gamma (q_0)$ from [26, Definition 5.1]. Let *T* be a bounded operator on $L^p$ for $p \in (p_0,q_0)$.

#### Definition 7.2

For $$q_0 < \infty $$ define

$$\begin{aligned} \gamma (q_0) :=\sup \big \{\gamma \ge 0 \,\vert \, \forall \epsilon > 0, \limsup _{p \rightarrow q_0}(q_0 - p)^{\gamma - \epsilon } \Vert T \Vert _{L^p\rightarrow L^p} = \infty \big \} , \end{aligned}$$

and for $$q_0 = \infty $$

$$\begin{aligned} \gamma (\infty ) :=\sup \big \{ \gamma \ge 0 \,\vert \, \forall \epsilon >0, \limsup _{p\rightarrow \infty } \frac{\Vert T \Vert _{L^p\rightarrow L^p}}{p^{\gamma -\epsilon }} = \infty \big \}. \end{aligned}$$

We say that an operator *T* admits a $(p_0,q_0)$ quadratic sparse domination if it satisfies a bound as the one in Theorem 1.7. We have the following upper bound on the unweighted norm of *T*.

#### Proposition 7.3

Let $q^* :=(q/2)'$. If *T* admits a $(p_0,q_0)$ quadratic sparse domination then for $p>2$ we have

$$\begin{aligned} \Vert T \Vert _{L^p\rightarrow L^p} \lesssim \left[ \left( \frac{p}{p_0}\right) '\right] ^{\frac{1}{p_0}} \left[ \left( \frac{p^*}{q_0^*}\right) '\right] ^{\frac{1}{2} \frac{1}{q_0^*}} \end{aligned}$$

and in particular

7.1.1 $$\begin{aligned} \gamma (q_0) \le \frac{1}{2{q_0^*}}. \end{aligned}$$

#### Proof

As in [26, Remark 3.4], let ${\mathcal {S}}$ be a $\eta$-sparse family. For $p>2$ we havewhere the last inequality follows from the bound on the $L^p$-norm of ${\mathcal {M}}^{\mathscr {D}}$ in (2.1.1), since

$$\begin{aligned} \Vert {\mathcal {M}}^{\mathscr {D}}_{\frac{p_0}{2}}(|f|^2 )\Vert _{L^{p/2}} = \Vert {\mathcal {M}}^{\mathscr {D}}(|f|^{p_0}) \Vert _{L^{p/p_0}}^{2/p_0} . \end{aligned}$$

$$\square $$

#### Remark 7.4

The upper bound on $\gamma (q_0)$ in (7.1.1) implies that, if $\gamma (q_0)$ equals $1/(2q_0^*)$ then the weighted estimates (1.0.6) are sharp.
